# Dissecting cancer heterogeneity based on dimension reduction of transcriptomic profiles using extreme learning machines

**DOI:** 10.1371/journal.pone.0203824

**Published:** 2018-09-14

**Authors:** Kejun Wang, Xin Duan, Feng Gao, Wei Wang, Liangliang Liu, Xin Wang

**Affiliations:** 1 College of Automation, Harbin Engineering University, Harbin, China; 2 Department of Biomedical Sciences, City University of Hong Kong, Hong Kong; The University of Texas MD Anderson Cancer Center, UNITED STATES

## Abstract

It is becoming increasingly clear that major malignancies such as breast, colorectal and gastric cancers are not single disease entities, but comprising multiple cancer subtypes of distinct molecular properties. Molecular subtyping has been widely used to dissect inter-tumor biological heterogeneity, in relation to clinical outcomes. A key step of this methodology is to perform unsupervised classification of gene expression profiles, which, however, often suffers challenges of high-dimensionality, feature redundancy as well as noise and irrelevant information. To overcome these limitations, we propose ELM-CC, which employs hidden observation features obtained from extreme learning machines (ELMs) for cancer classification. To demonstrate the effectiveness and usefulness, we applied ELM-CC for gastric and ovarian cancer subtyping. Comparing with the widely-used consensus clustering method, our approach demonstrated much better clustering performance and identified molecular subtypes that are much more clinically relevant.

## Introduction

Major malignancies have been demonstrated to be molecularly heterogeneous, which underlies the diverse clinical outcomes. In many cancers, heterogeneity exists both within the same tumor (intra-tumor heterogeneity) and across individual patients (inter-tumor heterogeneity) of the same histopathological type [1]. Therefore, cancers are no longer considered as single disease entities even in the same organ. Understanding the biological properties that distinguish tumors into molecular subtypes is a critical step for individualized therapy and design of more targeted agents. However, in the clinic cancer diagnosis, prognosis, and treatment decisions are still largely based on histopathological and clinical characteristics. For instance, tumors are classified according to tumor size, grade, disease stage, etc., which has proven prognostic values but poor predictive performance of drug efficacy due to a lack of clear molecular basis. Moreover, classifications based on molecular characteristics, e.g., *KRAS* mutation in colorectal cancer, have some predictive power, but still leave much of additional cancer heterogeneity unaccounted for [2].

The last decades have seen tremendous improvements of high-throughput technologies such as microarray and next-generation sequencing, which made it possible to more efficiently and cost effectively profile the expression levels for tens of thousands of genes in parallel. A gene expression profile can be denoted by a real-valued expression matrix, where rows and columns represent genes and samples, respectively. Clustering of gene expression profiles is a powerful approach to better dissect gene functions, gene regulatory mechanisms and molecular subgroups. A number of clustering approaches can be applied/developed for identification of biologically distinct subgroups. Generally, these clustering methods can be separated two major classes: partitional clustering algorithms and hierarchical clustering algorithms [3]. Commonly used methods such as *k*-means [4] and hierarchical [5] algorithms where objects are partitioned based on a (dis)similarity metric are the root algorithms upon which many new algorithms are built. Graph-based clustering algorithms [6] like spectral clustering regard gene expression data as a complete graph. Hence, clustering in this case becomes a graph partitioning problem. Partitioning Around Medoids (PAM) [7] and Self-Organizing Map (SOM) [8], which are flat clustering approaches, have also been widely used in gene clustering. These methods are straightforward and easy to use, but generally only for clustering of either genes or conditions/samples. To group simultaneously genes and conditions, Biclustering[9] and its extensions were proposed to mine gene clusters with respect to a subset of conditions from gene expression profiles. In special applications, more complicated clustering methods were developed, including Information-criterion based clustering algorithm[10], adaptive clustering [11], artificial neural networks[12]and ensemble clustering [13]. Furthermore, clustering methods have also been developed for integrative clustering of multi-omic data, represented by a joint latent variable model iCluster proposed by Shen et al. [14].

All the above-mentioned clustering methods have been demonstrated for their usefulness and effectiveness in diverse applications. The biggest drawback, however, lies in the arbitrary selection of the optimal number of clusters. To address the issue facing traditional clustering methods, consensus clustering methods have been developed [15].The original consensus clustering algorithm first performs subsampling of samples (or genes), and for each subsample runs conventional clustering (e.g. k-means). For varying cluster numbers, the algorithm subsequently calculates consensus values, which are the proportion that two samples are placed in the same cluster out of the total number of times they appear in the same subsamples. The area under the empirical cumulative distribution (CDF) curve is then calculated and define the optimal cluster count[15]. Instead, NMF-based consensus clustering performs matrix factorization to decompose the matrix of gene expression profiles to a small number of metagenes, each of which is a positive linear combination of all genes. For selection of the optimal number of clusters, NMF-based consensus clustering calculates cophenetic coefficients, which are a measure of dispersion of the consensus matrix [16]. Apart from the area under the CDF curve and cophenetic matrix, Gap statistic [17], a measure of within-cluster dispersion, can also be calculated for determination of the optimal number of clusters[18].

Recent advances in functional genomics applied to cancer biology are transforming the way cancers are traditionally categorized. One efficient strategy to dissect inter-tumor heterogeneity is to classify tumor samples into molecular subgroups using an unsupervised classification approach [19]. This approach takes as input gene expression profiles of primary tumor samples, and performs unsupervised classification to identify molecularly distinct subgroups, followed by biological and clinical characterizations and validations with independent data sets. Since whole-transcriptome expression data is employed, cancer classification obtained using this strategy has a strong molecular basis and a global functional landscape. Moreover, this approach is different from supervised classification incorporating prior clinical information such as survival and drug response, which is difficult to generalize and often leads to a biased conclusion. Therefore, unsupervised classification based on whole transcriptomic profiles has been widely applied for cancer subtyping, which has substantially improved our understanding about cancer heterogeneity and underlying subtype-specific biological mechanisms [20].

**Fig 1:**
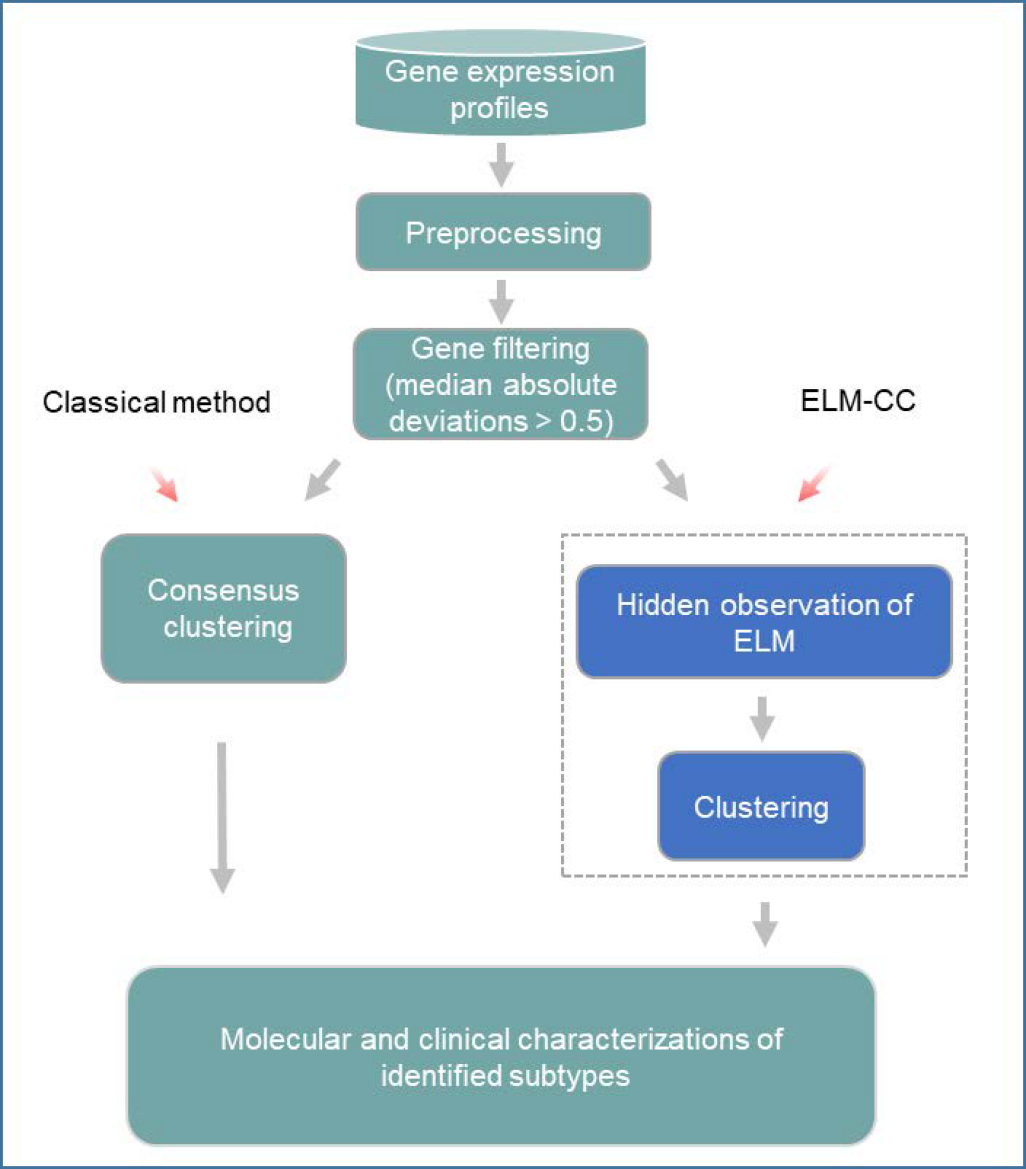
A schematic figure comparing the classical method and our new method for cancer molecular subtyping. The classical workflow involves several key steps (colored in cyan), whereas ELM-CC replaces the consensus clustering step by dimension reduction using an ELM followed by clustering (colored in blue).

A typical workflow of the above-mentioned approach for cancer classification involves multiple major bioinformatic steps (Fig 1). First of all, gene expression profiles for cancer samples obtained from high-throughput platforms such as microarrays are normalized using standard tools such as RMA, MAS5 and fRMA [20]. For RNA-Seq data, RPKM (Reads Per Kilobase of transcript per Million mapped reads) or TPM (Transcripts Per kilobase Million) data can be calculated using Tophat/Cufflinks[21] or RSEM [22] after quality control and alignment. Non-biological batch effects can be diagnosed by hierarchical clustering or principal component analysis, and can be corrected using popular tools such as ComBat [23]. Second, genes of low variability across tumor samples are not informative, and therefore, are filtered out before the following unsupervised classification. Third, expression levels for selected genes of high variability are subjected for clustering analysis, which seeks to detect the inherent biological differences and relationships between tumor samples.

The current popular method performs ‘consensus clustering’ [24]on the preprocessed gene expression data directly, which often suffers from several known drawbacks: 1) Even after feature selection, there are still thousands of genes retained, many of which are highly correlated and redundant. The redundancy noise and irrelevant features may drown the truly useful information and reduce the eventual clustering performance. 2) The clustering algorithms employed are also challenged by the high-dimensionality issues and the complexity of molecular data. 3) Consensus clustering performs bootstrap resampling of genes or samples, which can also be a concern especially for a small sample size.

To overcome the limitations of the abovementioned classical approaches, we propose ELM-CC, an extreme learning machines-based method for cancer classification [25]. An ELM is a feedforward neural network with a single layer of hidden nodes for classification or regression. ELMs can be used for dimension reduction and are featured with a fast learning speed and good generalization performance, compared to other approaches such as principal component analysis (PCA) and non-negative matrix factorization (NMF). Instead of direct clustering on high-dimensional gene expression data, we first train an ELM and take the observations at the hidden layer, with only a small number of nodes, for subsequent clustering analysis. To demonstrate the effectiveness of our ELM-CC, two real case studies on gastric cancer and ovarian cancer subtyping were carried out. Comparing with the classical approach based on consensus clustering, ELM-CC identified cancer subtypes that are much more molecularly distinct and clinically relevant.

## Methods

### Gene expression data sets

In the two case studies about gastric and ovarian cancer subtyping (details in Results), we used high-throughput gene expression profiles obtained from The Cancer Genome Atlas (TCGA) project as the training datasets. The TCGA gene expression data for gastric and ovarian were downloaded from Broad GDAC Firehose portal (http://gdac.broadinstitute.org/, accessed on Oct 1, 2016).

For gastric cancer, level-3 RNA-Seq data (n = 415) based on Illumina HiSeq platform were downloaded, which contains expression levels of 20,531 genes (S1 Table). Scaled estimates in the gene-level RSEM files were first converted to TPM (transcripts per million) by multiplying with 106 and then log2-transformed. Out of the total 415 patient samples, 200 have corresponding disease-free survival (DFS) information, which was used for survival analysis.

For ovarian cancer, normalized gene expression profiles (n = 514) based on Affymetrix U133A microarray platform, which contains expression levels of 10,771 genes, were downloaded and used directly in processed form (S1 Table). In total, overall survival (OS) information for 511 patients are available and were used for survival analysis.

To further evaluate the performance of our approach, the trained classifiers were applied to two independent data sets from Gene Expression Omnibus (GEO) database. For gastric cancer, GSE26253[26](n = 277) was used and this data set was measured on Illumina HumanRef-8 WG-DASL v3.0 platform, containing 17,418 genes. The expression data was processed using Genomestudio and the processed form was obtained using bioconductor package ‘GEOquery’ in R. For ovarian cancer, we adopted GSE26712[27] (n = 185) as the independent validation cohort. This data set was measured on Affymetrix Human Genome U133A Array microarray platform, containing 22,283 genes. Robust Multi-Array Analysis (RMA) was used to process the data and the processed form was also accessed in R using ‘GEOquery’ package.

### Molecular subtyping using the classical consensus clustering approach

The bioinformatic workflow employed by the classical approach for cancer subtyping involves major steps of data preprocessing, consensus clustering and classification.

The pre-processing step mainly includes data normalization, filtering of non-informative genes and removing potential batch effects. Since level-3 gene expression data from TCGA were already normalized, we used median absolute deviation (MAD), a summary statistic indicating dispersion, to filter out genes with low variability. Subsequently, consensus clustering was performed on the preprocessed gene expression profiles, aiming to identify the optimal number of clusters [8]. Consensus clustering has been successfully applied for molecular subtyping for various cancer types [19]. More specifically, it first performs subsampling of patient samples (or genes) and then runs a conventional clustering algorithm on each subsample. This step is repeated for thousands of times, and a consensus matrix is calculated in order to assess the robustness and stability of clustering. Each value in the consensus matrix is the proportion that two samples are placed in the same cluster out of the total number of times they appear in the same subsamples. The area under the empirical cumulative distribution (CDF) curve is then calculated for identification of the optimal cluster count. The consensus clustering algorithm used in our case studies was implemented in R package ‘ConsensusClusterPlus’ with the default parameter setting.

### ELM algorithm for dimension reduction of transcriptomic profiles

Extreme learning machine (ELM) was initially proposed in 2006 [25]. An ELM is a single-hidden layer feedforward neural network (SLFN), which randomly chooses hidden nodes and analytically determines the output weights. Comparing with traditional neural networks which adopt gradient-based learning algorithms for training, extreme learning machines provide good generalization performance at an extremely fast learning speed. In this paper, we propose to use ELM observations at the hidden layer, named an “ELM feature matrix”, obtained from regression analysis of gene expression profiles for cancer classification.

**Fig 2:**
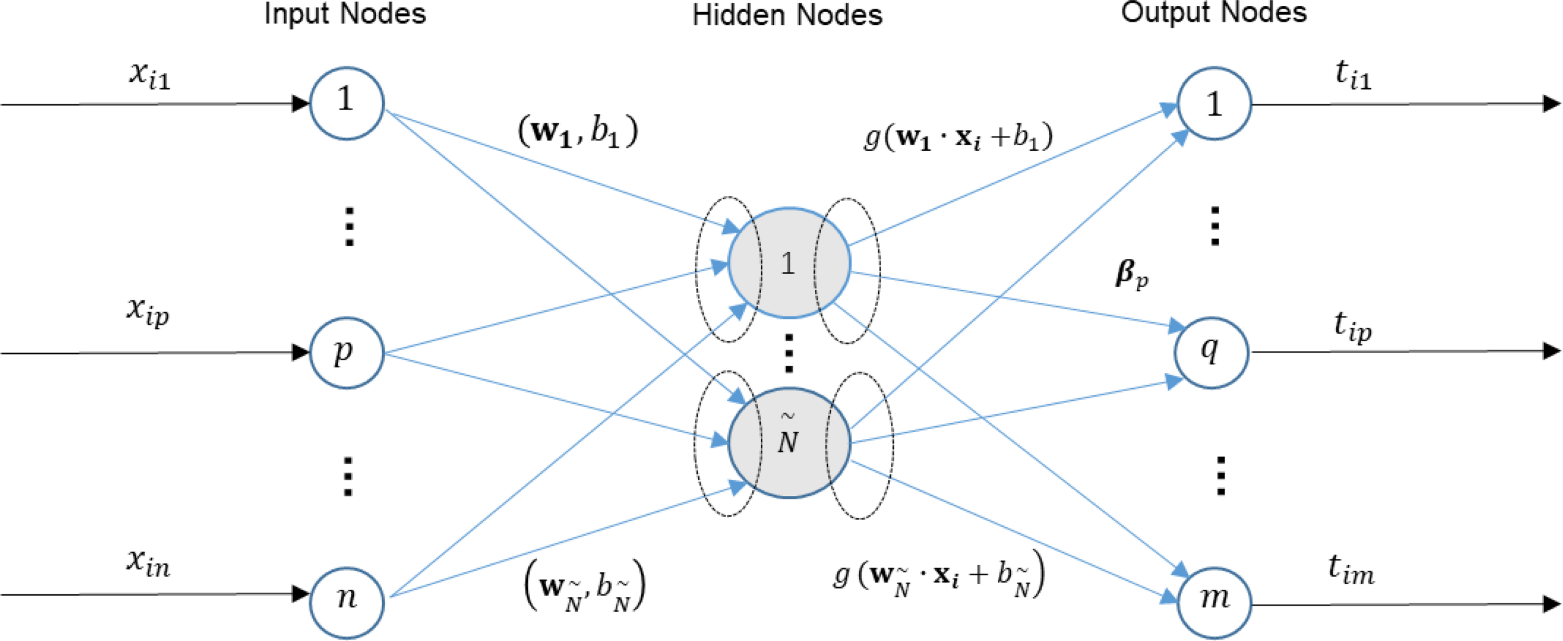
A schematic figure illustrates an extreme learning machine for regression.

Fig 2 illustrates the principle of an ELM for regression, and how an ELM feature matrix can be extracted from a single hidden layer feedforward network (SLFN).

In the ELM regression model, the number of neurons at the input layer and output layer are both equal to the number of samples. Given *N* cancer samples (**x**_*i*_,**t**_*i*_), where **x**_*i*_ = [*x*_*i*1_,*x*_*i*2_,…,*x*_*in*_] ∈ *R*^*n*^ is the input data and **t**_*i*_ = [*t*_*i*1_,*t*_*i*2_,…,*t*_*im*_]^*T*^ ∈ *R*^*m*^ is the target data, *i* = 1,…,*N*. For regression, the input data is also the target data. Define a standard SLFN with $\tilde{N}$ hidden nodes, which is also the dimension of expected feature space, and an activation function *g*(*x*). The standard SLFN is modeled as:

$$\sum_{i=1}^{\tilde{N}}\beta_{i}g_{i}(x_{j})=\sum_{i=1}^{\tilde{N}}\beta_{i}g({w_{i}∙x}_{j}+b_{i}),\;j=1,\ldots ,N.$$ (1)

where **w**_*i*_ = [*w*_*i*1_,*w*_*i*2_,…,*w*_*in*_]^*T*^ is the weight vector between the *i*th hidden node and input nodes, and **β**_*i*_ = [*β*_*i*1_,*β*_*i*2_,…,*β*_*im*_]^*T*^ is the weight vector connecting the *i*th hidden node and the output nodes. *b*_*i*_ is the bias of the *i*th hidden node. Huang et al.[25] proved there exists **β**_*i*_, **w**_*i*_ and *b*_*i*_, such that

$$\sum_{i=1}^{\tilde{N}}\beta_{i}g({w_{i}∙x}_{j}+b_{i})=t_{j},\;j=1,\ldots ,N.$$ (2)

The SLFN can be trained by finding a least-square solution of Eq (2).

After training the SLFN, we obtain a feature matrix F defined as follows:

$$F=[\begin{array}{c} w_{1}{∙x}_{1}{+b}_{1}\cdots w_{\tilde{N}}{∙x}_{1}{+b}_{\tilde{N}}\\ \vdots \\ w_{1}{∙x}_{N}{+b}_{1}\cdots w_{\tilde{N}}{∙x}_{N}{+b}_{\tilde{N}} \end{array}{]}_{N\times \tilde{N}}$$ (3)

ELM essentially performs random projection of high-dimensional data onto a low-dimensional feature space. Random projection is a general data reduction technique and has been demonstrated for its promising performance [28]. ELM feature can preserve well the similarity of data vectors, while the dimensionality can be dramatically reduced from a space of thousands of genes. Moreover, computationally ELM is significantly less expensive than other methods such as principal component analysis and non-negative matrix reduction.

### ELMs for classification

Having obtained ELM feature matrix, conventional clustering algorithms such as *k*-means can be used for cancer classification. An ELM can also be trained as a classifier, where the number of output nodes is set to the number of cancer subtypes. Unlike the ELM regression model for feature training, the activation function in the ELM classifier is set to Hard-Limit transfer function, which allows a neuron to make a decision for classification. In either case study, we built an ELM classifier using the training dataset, and validated the performance using an independent dataset.

### Statistics

Statistical analyses were performed using R (version 3.4.3, www.r-project.org). Survival analyses were performed using the Kaplan-Meier method and compared using a log-rank test by ‘survival’ package. Multivariate cox regression models were trained using ‘coxph’ function in ‘survival’ package. Hazard ratios were calculated using function ‘hazard.ratio’ in ‘survcomp’ package. P < 0.05 was considered as significant for all tests. Differential gene expression analysis was performed for each subtype versus the other subtypes identified by ELM-CC using ‘limma’ R package. Gene set enrichment analysis (GSEA) was performed using ‘HTSanalyzeR’ package.

## Results and discussion

### Case study in gastric cancer

Gastric cancer (GC), also known as stomach adenocarcinoma, is one of the major malignancies and the second leading cause of cancer-related death [29]. Previous studies showed GC is a molecularly heterogeneous disease, but how to define the subtypes remains controversial [30–31]. More importantly, none of previous studies have demonstrated a significant association with disease-free survival (DFS), which is an important factor of clinical relevance.

We first applied the classical consensus clustering approach to classify stomach adenocarcinoma (STAD) samples obtained from TCGA (*n* = 415). At the preprocessing step, we filtered out genes with low variability quantified by median absolute deviations (or MAD). Out of the total 20,531 genes, 3,245 were kept after filtering (MAD > 1). Consensus clustering was subsequently performed with bootstrap resampling using R package ‘ConsensusClusterPlus’ [24]. Four robust clusters were identified to be the optimal (S1 Fig), as the area under the empirical cumulative distribution function (CDF) did not increase substantially (< 0.1) from 4 to 5 clusters, and so on (S2 Fig). In addition to the empirical cumulative distribution function (CDF), we used gap statistic [17] to determine the optimal clustering number. More specifically, we calculated gap statistic using the ELM hidden feature of gastric cancer for *k* = 2 to 6, and a peak was found at *k* = 4 (S3A Fig). For a benchmark study, we also applied other classical algorithms such as *k*-means, hierarchical clustering, spectral clustering as well as non-negative matrix factorization (NMF) based approach to classify the same data set into four subtypes. Gap statistic was also performed on the preprocessed gene expression data, and the result indicates that the optimal number of clusters is *k* = 4 (S3B Fig).

As a comparison, we next employed an ELM for regression using R package ‘elmNN’, taking as input the preprocessed gene expression profiles. Since the ELM was used for dimensionality reduction, the number of the hidden nodes was deliberately set to $\tilde{N}=3$, which also facilitate the visualization. After ELM fitting, we calculated hidden observation features (S2 Table) using formula (3), which was then subjected for clustering using *k*-means algorithm (*k* = 4) [4].

**Fig 3:**
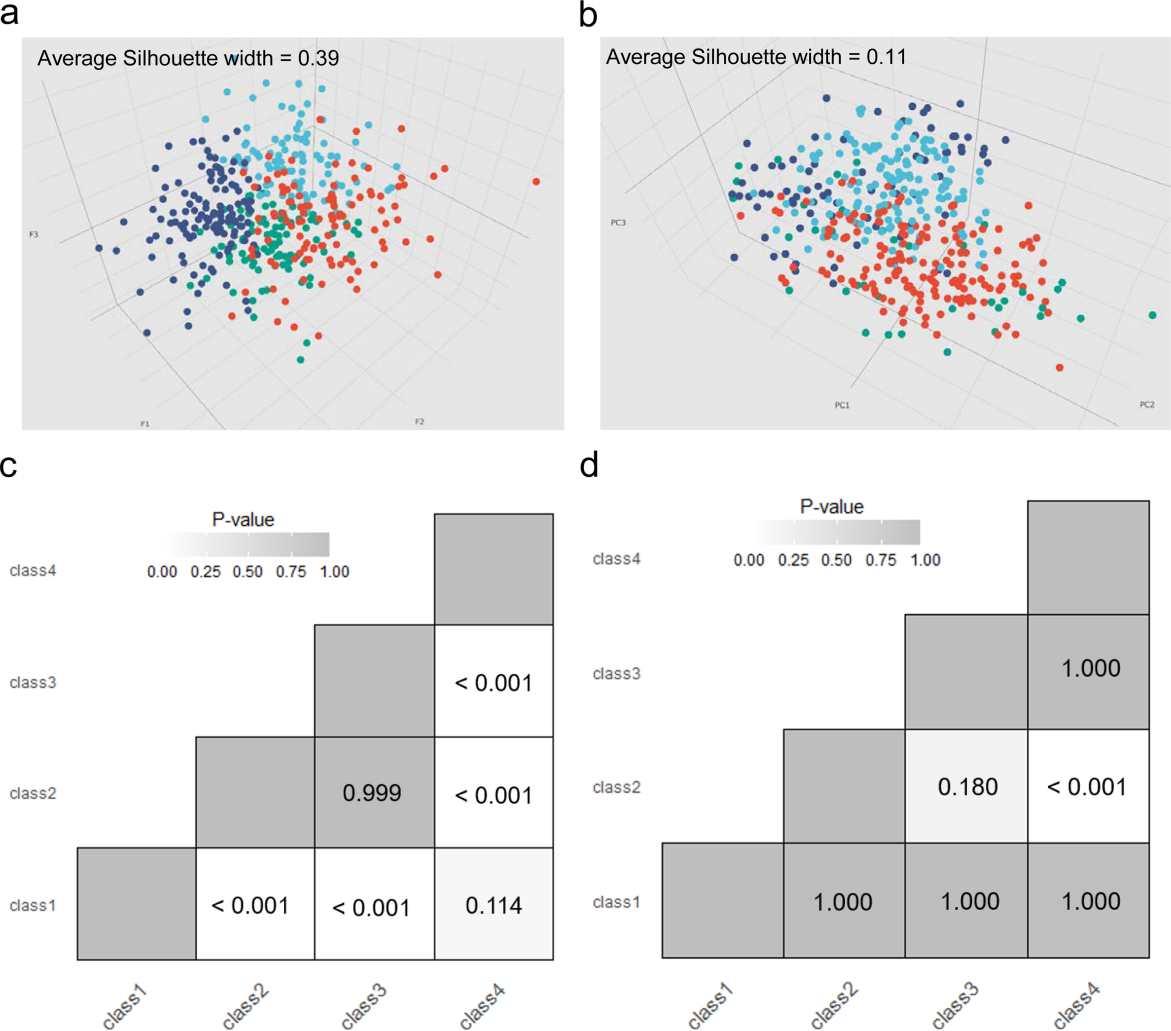
Benchmark of clustering performance in the gastric cancer subtyping case study. 3D visualization revealed patient samples classified to different subtypes were much more tightly distributed in (a) the three-dimensional feature space based on ELM-CC clustering result (average Silhouette width = 0.39) than (b) in the space of top three principal components of gene expression profiles based on Consensus clustering result (average Silhouette width = 0.11). SigClust analysis showed more statistically significant (*P* < 0.05) pairwise comparisons of subtypes identified based on (c) ELM-CC than (d) the counterpart based on classical consensus clustering method.

We compared the visualizations of samples in the three-dimensional space based on the three ELM hidden features (F1-F3) and the first three principal components (PC1-PC3) calculated from the preprocessed gene expression profiles. Interestingly, patient samples classified to different subtypes are much more tightly distributed in the three-dimensional feature space based on ELM-CC (Fig 3). In contrast, cancer subtypes identified using the classical workflow (Consensus clustering) do not show clear boundaries (Fig 3B).

To quantitatively benchmark the clustering performance, we employed statistical significance of clustering (SigClust) [32] to evaluate clustering performance, which is an approach to test the statistical significance of clusters in high-dimensional data. To make fair comparison, for both ELM-CC and the classical approach we took as input the same gene expression data used for clustering analysis. SigClust analysis showed that 4 of the total 6 pairwise comparisons of the subtypes identified by ELM-CC were significant (*P* < 0.05, Fig 3C), whereas only 1 pairwise comparison was significant for the classical approach (Fig 3D).

**Fig 4:**
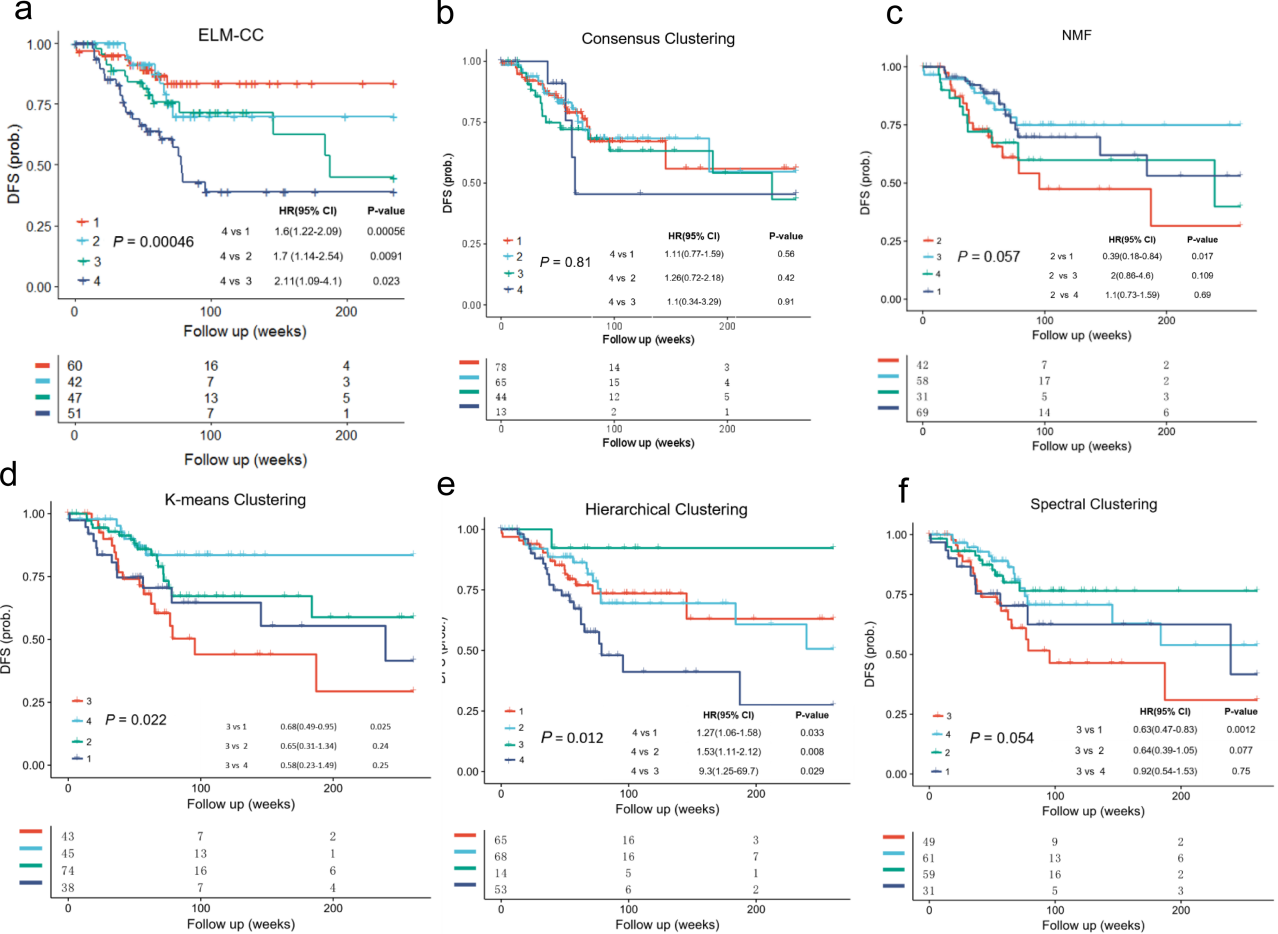
**Kaplan-Meier plots compare the associations of molecular subtypes of gastric cancer identified using (a) ELM-CC, (b) Consensus clustering, (c) NMF clustering, (d) *k*-means clustering, (e) hierarchical clustering and (f) spectral clustering with disease free survival (DFS)**.

To further evaluate the clinical relevance of our clustering results, we performed survival analyses. Disease-free survival (DFS) is the length of time that patient survives with no symptoms after the treatment for a cancer, which is a common factor to indicate the different clinical outcomes across groups. The subtypes identified using ELM-CC showed a much more significant association with DFS (Fig 4A, *P* = 0.00046, log-rank test) than the counterparts based on the other classical clustering algorithms (Fig 4B–4F, *P* > 0.01, log-rank tests). In addition, previous study [30] showed no significant differences in DFS (*P* = 0.068, log-rank test) (S4 Fig). Using TCGA gastric cancer data set for training, we constructed an ELM classifier and then applied to an independent cohort GSE26253 (n = 277) and the classification results also showed significant association with DFS (S5 Fig). To investigate whether the subtypes identified by ELM-CC show consistent association with survival, we performed Cox proportional hazards analyses for pairwise comparisons between different subtypes. Indeed, we found class 4 is associated with worse prognosis than class 1–3 consistently in both the training (Fig 4A) and validation (S5 Fig) data sets.

To compare ELM-CC clustering with other clinical risk factors, we performed univariate and multivariate Cox regression analyses of covariates including patient age (> = 65 vs < 65), sex (male vs female), tumor stage (III-IV vs I-II), lymph node (LN) metastasis (positive vs negative), consensus clustering (class 4 vs classes 1–3) as well as ELM-CC clustering (class 4 vs classes 1–3). In both univariate and multivariate analyses, ELM-CC classification is the factor that is most significantly associated with patient survival (Table 1). Stage and lymph node metastasis state are also significant in the univariate analysis (*P* = 0.003 and 0.01, respectively) but lost their significant associations in the multivariate analysis (Table 1). Consensus clustering is not significant in both the univariate and multivariate analyses (Table 1).

**Table 1:** Univariate and multivariate analyses for gastric cancer.

	Univariate analysis		Multivariate analysis	
	HR (95% CI)	p value	HR (95% CI)	p value
Age (> = 65 vs. <65)	1.20 (0.70~2.04)	0.51	1.57 (0.91~2.71)	0.11
Sex (male vs. female)	1.85 (0.97~3.52)	0.06	1.88 (0.96~3.65)	0.06
Stage (III–IV vs. I–II)	2.46 (1.36~4.47)	0.003	1.23 (0.79~1.92)	0.35
LN (positive vs. negative)	2.44 (1.23~4.84)	0.01	1.76 (0.63~4.96)	0.28
Consensus Clustering (class 4 vs classes 1–3)	1.15(0.69~1.92)	0.59	1.34(0.80~2.26)	0.27
ELM-CC (class 4 vs classes 1–3)	1.67 (1.30~2.16)	<0.0001	1.64 (1.26~2.13)	<0.0001

**Fig 5:**
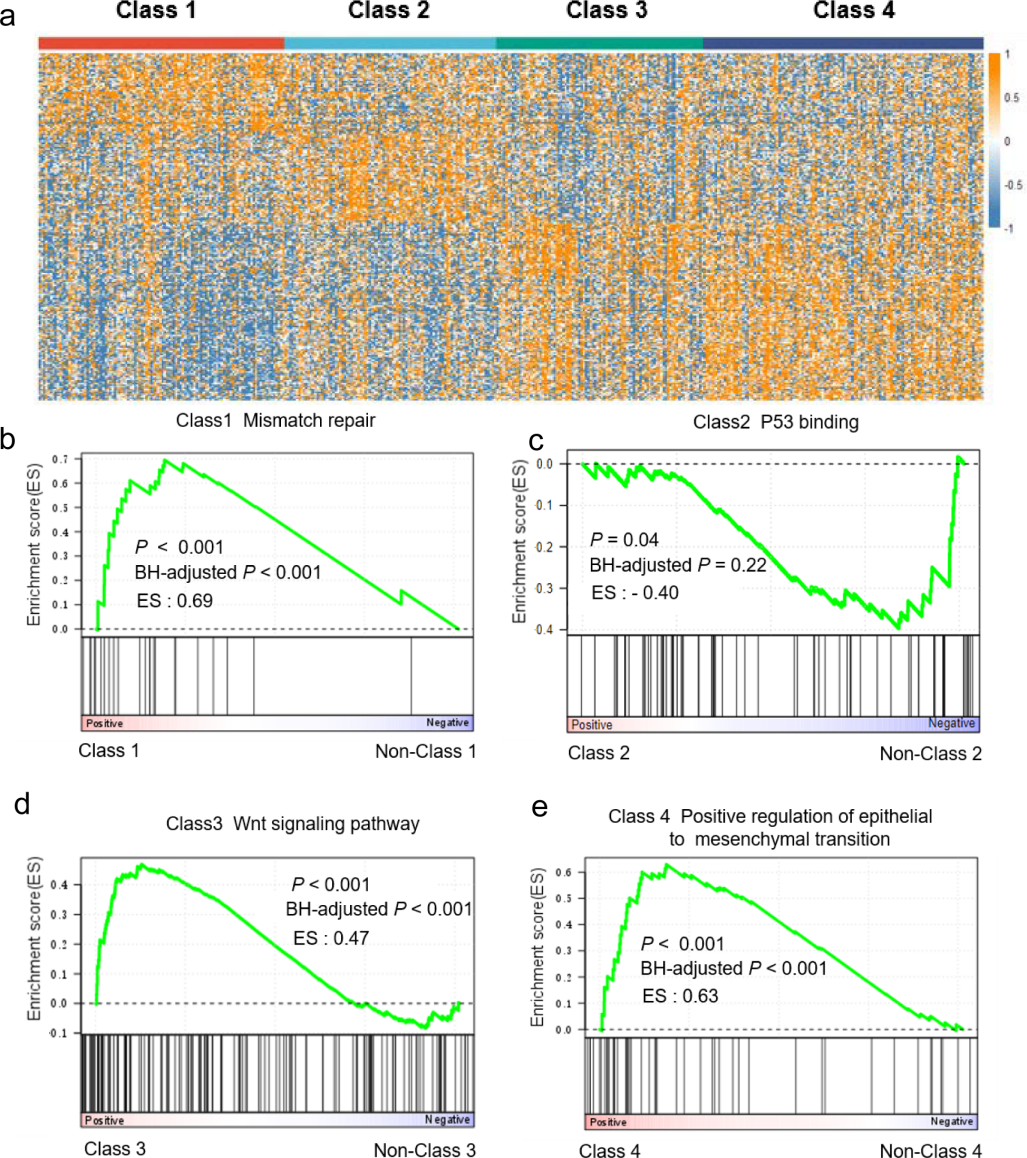
Differential gene expression and pathway analyses in gastric cancer. (a) Heatmap illustrating signature genes that are differentially expressed between identified clusters. (b-e) GSEA plot illustrating a representative pathway dysregulated in each molecular subtype identified.

To elucidate biological characterizations of identified subtypes, we analyzed differentially expressed genes in each subtype as compared to the others (Fig 5A) and then performed biological characterizations based on gene set enrichment analysis (GSEA) for identification of dysregulated pathways in each subtype. Top statistically significant pathways identified for each subtype (S4 Table) suggested that class 1 is featured with mismatch repair (Fig 5B); class 2 is characterized by dysregulated p53 pathway (Fig 5C); class 4 is characterized by activated epithelial to mesenchymal transition (Fig 5E). To further confirm p53 dysregulation in class 2, we calculated TP53 activity scores using a two-gene signature (CDKN1A and MDM2), which was employed previously for differentiating ‘TP53-’ from ‘TP53+’ subtypes [30]. Indeed, comparing with the other three classes, TP53 activity is significantly lower in Class 2 (*P* = 0.01, one-tailed Student’s *t* test) (S6 Fig). Based on the pathway analysis, class 1, 2 and 4 recapitulated the ‘MSI’, ‘TP53 -’, and ‘EMT’ subtypes previously reported [30]. Class 3 is associated with upregulated Wnt signaling pathway (Fig 5D), which is newly identified and was not reported before.

### Case study in ovarian cancer

Ovarian cancer is the fifth leading cause of cancer-related deaths among women, accounting for more deaths than any other cancers in the female reproductive systems. Ovarian cancer mainly develops in older women, and especially, women older than 65 are most affected by this type of cancer. Most of the ovarian cancer are carcinomas of the surface epithelial type [33], which accounts for the vast majority of all ovarian cancers. Similar to other major malignancies, ovarian cancer is already known to be heterogeneous, where multiple molecular subtypes exist and correlate with clinical outcomes [34–35].

Here, we sought to investigate whether ELM-CC is generally applicable, not only to gastric cancer, but also to other cancers. We performed similar analyses to what we have done for gastric cancer. We first applied the classical workflow to classify Ovarian serous cystadenocarcinoma samples obtained from TCGA (*n* = 514). Although previous studies on ovarian cancer subtyping attempts to use the TCGA data set as well, it is hard to obtain a reasonable subtyping result as indicated by many research groups [34]. After preprocessing, 2019 genes (MAD > 0.5) were kept for the following analyses. Consensus clustering identified four robust clusters (S7 Fig), as the area under the empirical cumulative distribution function (CDF) did not increase substantially (< 0.1) from 4 to 5 clusters, and so on (S8 Fig), which was also confirmed by gap statistic (S9B Fig). For a benchmark study, we also applied other classical algorithms such as *k*-means, hierarchical clustering, spectral clustering as well as non-negative matrix factorization (NMF) based approach to classify the same data set into four subtypes. Gap statistic was also performed on the preprocessed gene expression data, and the result indicates that the optimal number of clusters is *k* = 4 (S9B Fig).

**Fig 6:**
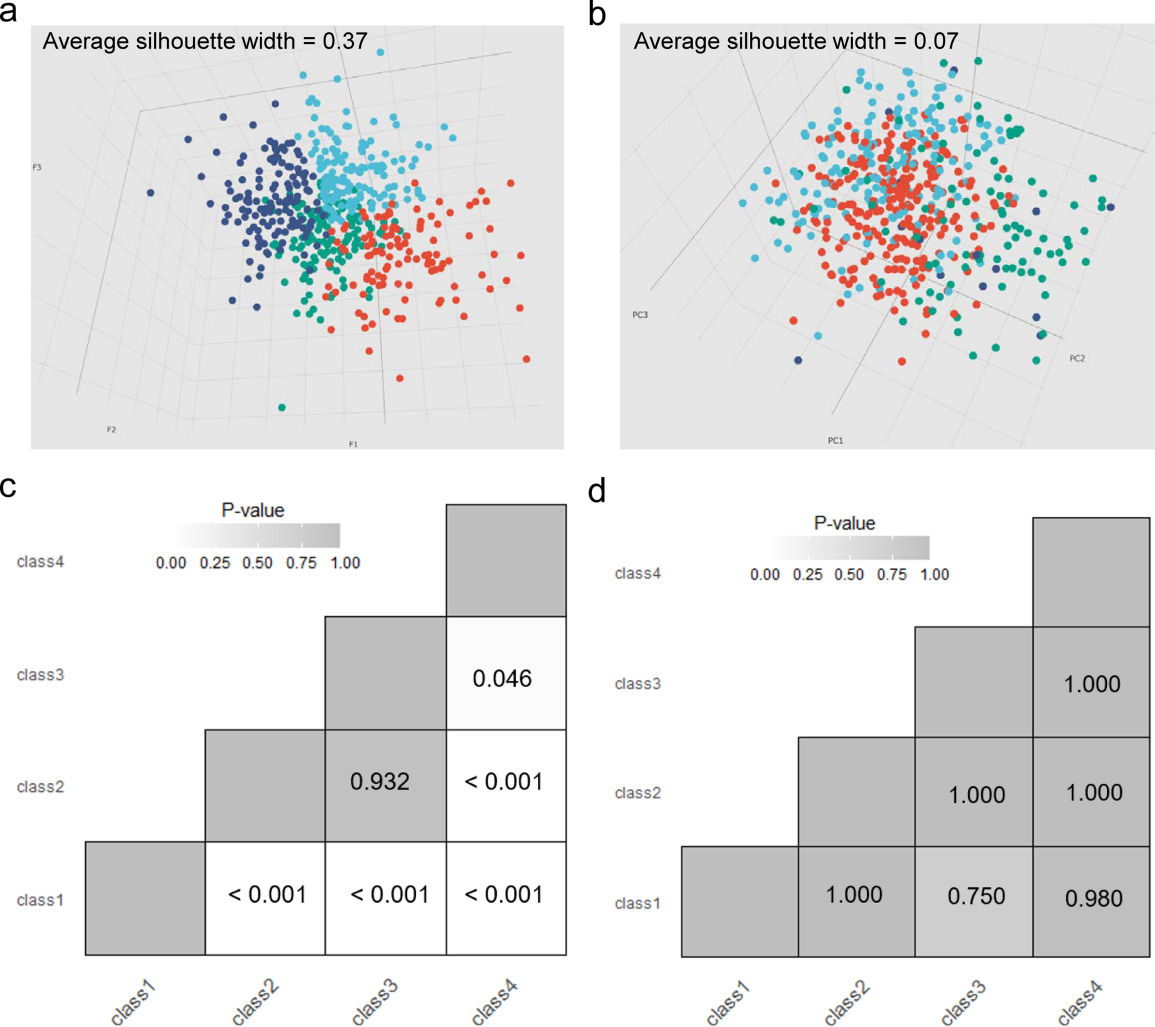
Benchmark of clustering performance in the ovarian cancer subtyping case study. 3D visualization revealed patient samples classified to different subtypes were much more tightly distributed in (a) the three-dimensional feature space based on ELM-CC clustering result (average Silhouette width = 0.37) than (b) in the space of top three principal components of gene expression profiles based on Consensus clustering result (average Silhouette width = 0.07). SigClust analysis showed more statistically significant (*P* < 0.05) pairwise comparisons of subtypes identified based on (c) ELM-CC than (d) the counterpart based on classical consensus clustering method.

We next employed an ELM for regression analysis using the preprocessed gene expression profiles. After ELM fitting, we calculated the feature matrix (S3 Table) using formula (3), which was then subjected for clustering using *k*-means algorithm. The optimal clustering number on the feature matrix using gap statistic is *k* = 4 (S9A Fig). 3D visualization showed that patient samples classified to different subtypes are much more tightly distributed in the three-dimensional feature space based on ELM-CC (Fig 6A–6B). Compared to consensus clustering, ELM-CC demonstrated much better clustering performance, as indicated by its more significant difference between subtypes using SigClust (Fig 6C–6D).

**Fig 7:**
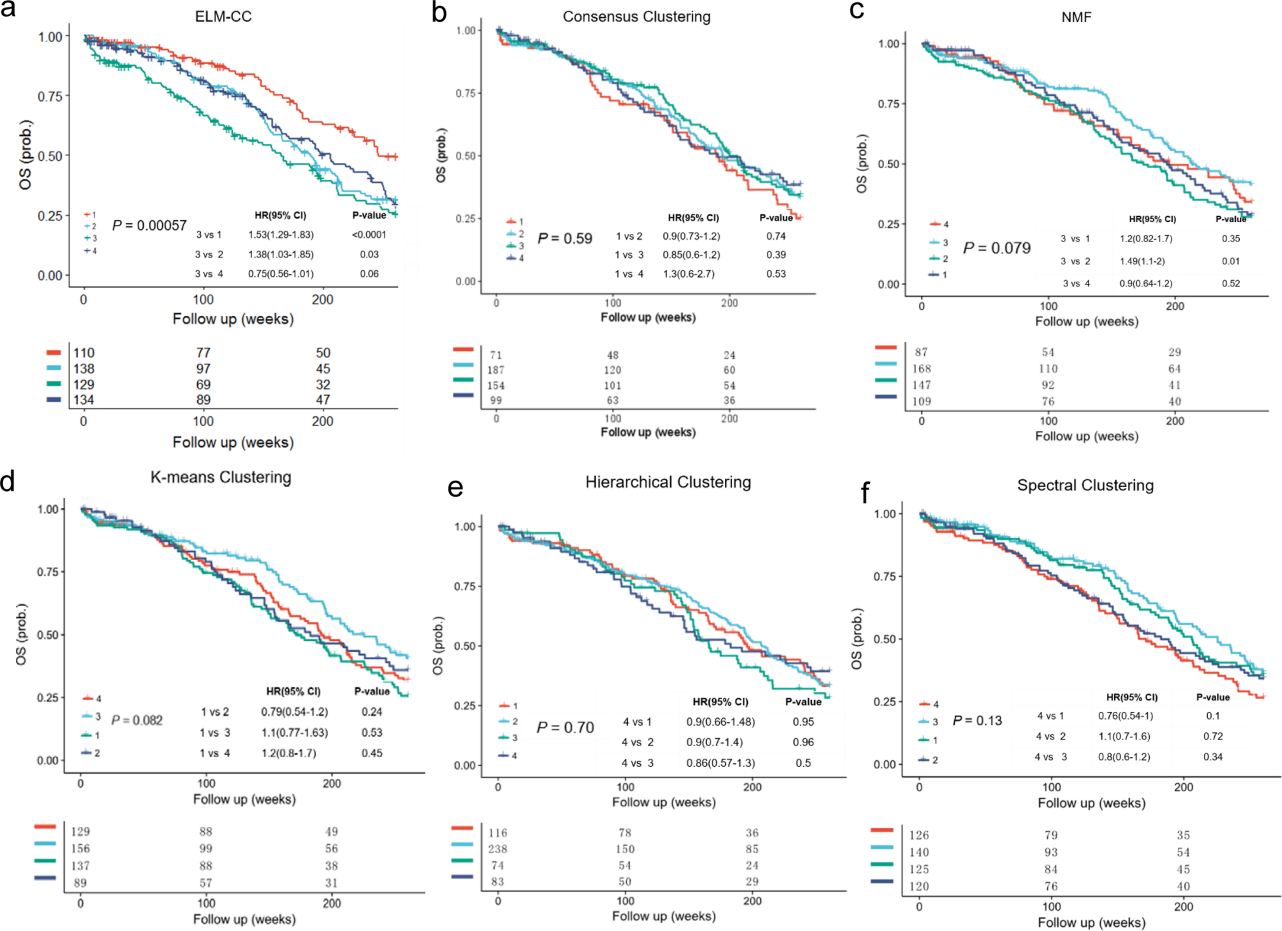
**Kaplan-Meier plots compare the associations of molecular subtypes of ovarian cancer identified using (a) ELM-CC, (b) Consensus clustering, (c) NMF clustering, (d) *k*-means clustering, (e) hierarchical clustering and (f) spectral clustering with overall survival (OS)**.

Furthermore, we performed survival analyses to evaluate the clinical association of the clustering results. For ovarian cancer, a common indicator is overall survival (OS), which is the length of time that patients diagnosed with the cancer are still alive from the diagnosis or the treatment start date. Difference in OS between different subgroups can indicate the distinct clinical outcomes, which leads to more optimized treatment in clinical practice. In contrast to the poor performance of the classical algorithms (*P* > 0.01, log-rank tests) (Fig 7B–7F) and previous study (*P =* 0.085, log-rank test) (S10 Fig) the identified four subtypes based on ELM-CC are significantly associated with OS (Fig 7A, *P* = 0.00057, log-rank test). Furthermore, the robustness was tested on a set of independent samples from GSE26712 (n = 185), which showed significant association with OS as well (*P* = 0.03, log-rank test) (S11 Fig). Univariate and multivariate analyses indicate that subtypes identified by ELM-CC provide a more significant predictor of overall survival, compared with other clinical characteristics (Table 2).

**Table 2:** Univariate and multivariate analyses for ovarian cancer.

	Univariate analysis		Multivariate analysis	
	HR (95% CI)	p value	HR (95% CI)	p value
Age (> = 65 vs. <65)	1.53 (1.21~1.93)	0.0002	1.41 (1.11~1.80)	0.006
Grade (high vs. low)	0.86 (0.61~1.21)	0.38	0.86 (0.59~1.26)	0.44
Stage (late vs. early)	2.43 (1.15~5.14)	0.021	2.24 (0.90~5.56)	0.08
Debulking (suboptimal vs. optimal)	1.50 (1.17~1.94)	0.0017	1.42 (1.09~1.84)	0.009
Consensus Clustering (class 1 vs classes 2–4)	0.94(0.75~1.18)	0.61	0.97(0.76~1.24)	0.82
ELM-CC (class 3 vs classes 1/2/4)	1.59 (1.24~2.04)	0.0002	1.68 (1.29~2.20)	0.0001

**Fig 8:**
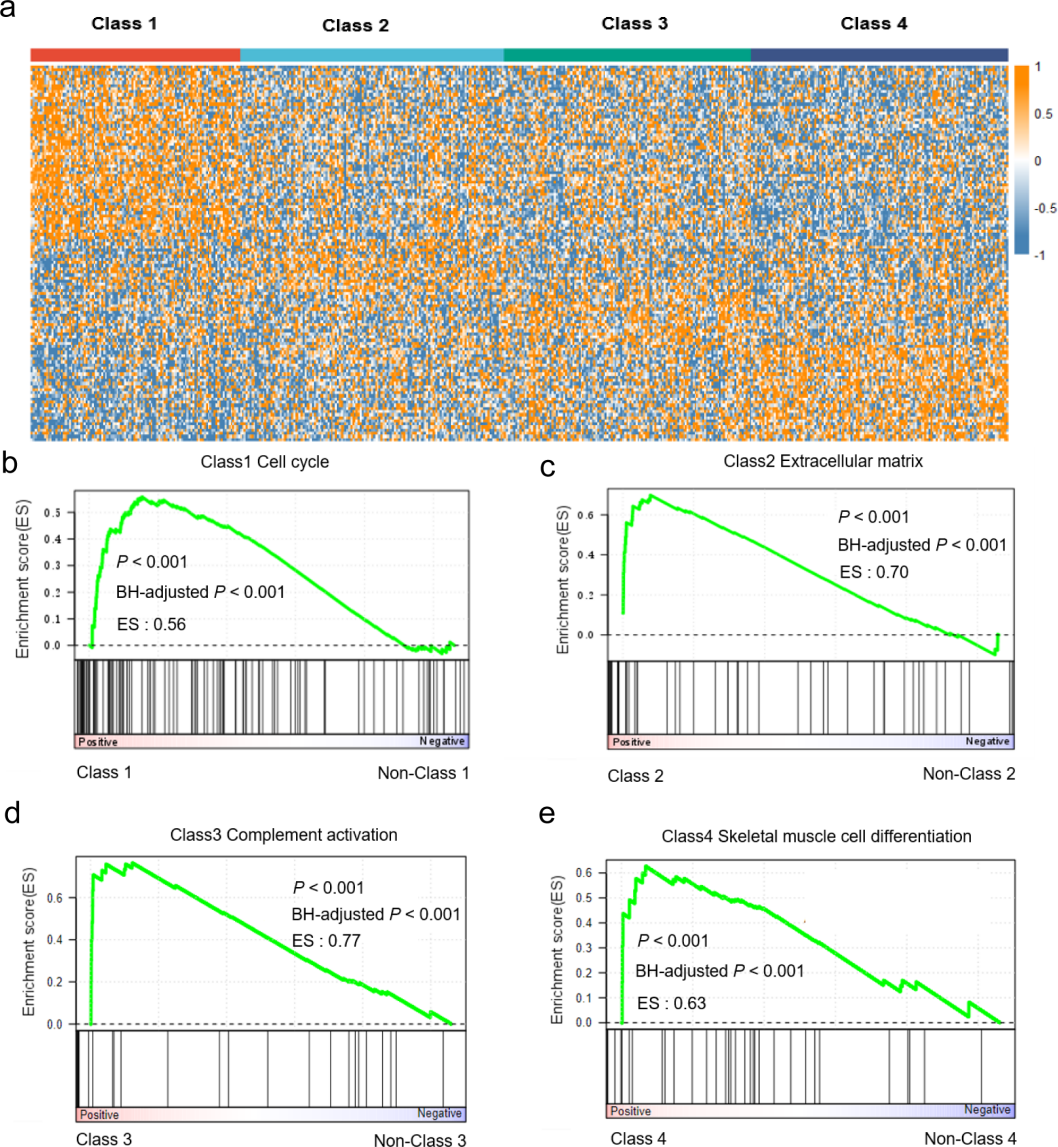
Differential gene expression and pathway analyses in ovarian cancer. (a) Heatmap illustrating signature genes that are differentially expressed between identified clusters. (b-e) GSEA plot illustrating a representative pathway dysregulated in each molecular subtype identified.

To elucidate biological properties associated with identified subtypes, we analyzed differentially expressed genes between subtypes (Fig 8A) and then performed biological characterizations based on gene set enrichment analysis (GSEA). Top statistically significant pathways identified for class 1–4 (S4 Table) suggested their strong associations with dysregulated cell cycle (Fig 8B), extracellular matrix (Fig 8C), component activation (Fig 8D) and cell differentiation related pathways (Fig 8F), respectively. These results (Fig 8, S4 Table) confirmed that classes 1–4 identified by ELM-CC successfully recapitulated the ‘Proliferative’, ‘Mesenchymal’, ‘Immunreactive’ and ‘Differentiated’ subtypes previously identified [35].

### Case study on other cancer types

**Fig 9:**
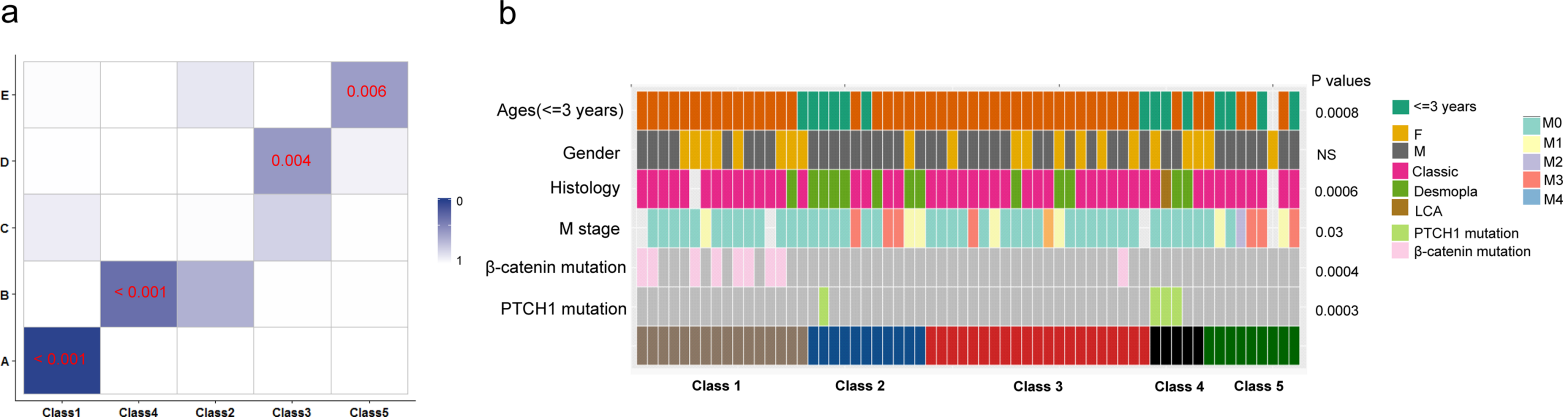
Identification of molecular subtypes in medulloblastoma by ELM-CC. (a) Hypergeometric tests showed high concordance between ELM-CC subtypes (Class 1–5) and the previously characterized subtypes (A-E). P-values were derived from Fisher’s exact tests. (b) Heatmap illustrating clinical and molecular characteristics for all 62 patients.

To further demonstrate the general applicability, we employed ELM-CC to dissect molecular heterogeneity of medulloblastoma and large B-cell lymphoma. For medulloblastoma, gene expression profiles for 62 samples published in *Kool et al* [36] were used to perform ELM-CC clustering after gene filtering (MAD > 0.5). The five subtypes identified by ELM-CC achieved a generally high concordance with the five subtypes (A, B, C, D, and E) identified by *Kool et al* [36] as indicated by significant p-values derived from hypergeometric tests for pairwise comparisons (Fig 9A). More interestingly, integrative analysis with mutations revealed that tumors classified to Class 1 were enriched for β-catenin mutations (*P* = 0.0004, Fisher’s exact test), which is a well-known characteristic for subtype A medulloblastoma. Class 4 tumors were enriched for *PTCH1* mutations (*P* = 0.0003, Fisher’s exact test), which is also consistent with previous finding that subtype B medulloblastoma is characterized by *PTCH1* mutation [37] (Fig 9B).

**Fig 10:**
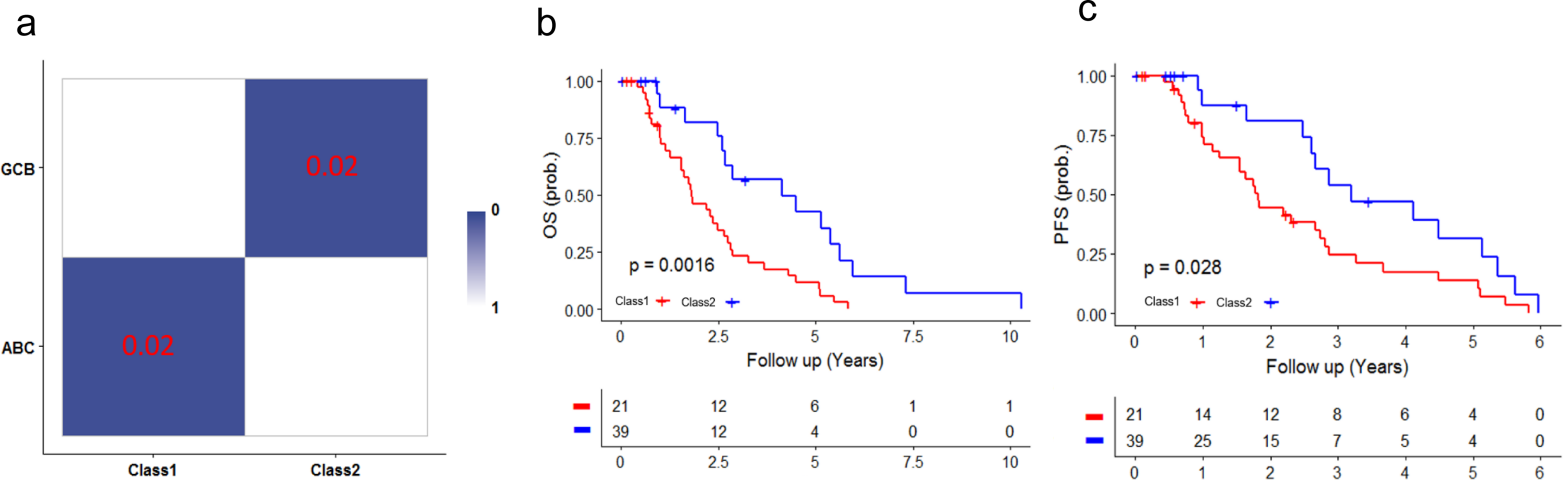
Identification of molecular subtypes in diffuse large B-cell lymphoma. (a) Hypergeometric tests showed high concordance between ELM-CC subtypes (Class 1–2) and the previously characterized subtypes (GCB and ABC). Kaplan-Meier plots showed significant associations of identified subtypes with (b) overall survival and (c) progression-free survival.

In the case study for diffuse large B-cell lymphoma (DLBCLs), we analyzed gene expression profiles for 60 patient samples obtained from *Shaknovich et al* [38]. Previous studies on DLBCLs have identified two major distinct subtypes: germinal-center B cell-like (GCB) and activated B cell-like (ABC) DLBCLs [38]. ELM-CC identified two subtypes significantly associated with GCB and ABC subtypes (*P* < 0.05) based on hypergeometric tests for pairwise comparisons (Fig 10A). Kaplan-Meier plots also showed significant association of identified subtypes with overall survival (Fig 10B, *P* = 0.0016, log-rank test) and progression-free survival (Fig 10C, *P* = 0.026, log-rank test).

Taken together, our case studies on various cancer types demonstrated that ELM-CC can not only recapitulate previously well characterized molecular subtypes, but also has a big potential to improve the performance by identifying subtypes that are more biologically coherent and clinically relevant.

## Conclusions

To summarize, our new approach ELM-CC incorporating features trained from extreme learning machine (ELM) can overcome the limitation of classical cancer subtyping method when dealing with high-dimensional transcriptomic data. Unlike other feature extraction methods (e.g. principal component analysis), ELM features are obtained based on random projection from high-dimensional input data onto a low-dimensional space, and the feature components are equivalent in every dimension. This brings strong robustness to ELM-CC to reveal the inherent molecular properties associated with different cancer subtypes. It should be noted, however, since an ELM essentially performs random projection of high-dimensional data onto a low-dimensional space, a known limitation is potential unstable performance especially for a small sample size due to the random initialization of ELM input weights. We demonstrated the superior performance of ELM-CC in two case studies for molecular subtyping of gastric cancer and ovarian cancer, respectively. Compared with previous studies by other research groups and the classical consensus clustering-based approach, the subtypes identified by ELM-CC showed much more clear clustering patterns and much stronger associations with survival. Although more comprehensive characterizations of the identified gastric and ovarian cancer subtypes are needed for gaining more mechanistic insights into the biology, which is our future work, ELM-CC has shown its potential for better dissection of cancer heterogeneity.

## Supporting information

S1 Fig Consensus matrix derived from consensus clustering in the classical workflow for the gastric cancer case study. (TIF)

S2 Fig Relative changes in area under the CDF curve in the gastric cancer case study, as the number of clusters (*k*) increase from 2 to 6. When *k* increases from 4 to 5 and so on, the area under the CDF curve does not increase substantially (<0.1), as indicated by the red line.
(TIF)

S3 Fig Gap statistic determined the optimal clustering number. The optimal clustering number is 4 using the ELM hidden feature(a) and preprocessed gene expression(b) for gastric cancer.
(TIF)

S4 Fig The four molecular subtypes defined in previous studies showed no significant association with disease-free survival in gastric cancer. (TIF)

S5 Fig Survival analysis based on classifications of the validation dataset showed significant association with DFS in gastric cancer. (TIF)

S6 Fig Class 2 gastric cancer subtype shows significantly lower TP53-activity score than the other classes. The TP53 activity score was calculated based on average expression levels of CDKN1A and MDM2 genes.
(TIF)

S7 Fig Consensus matrix derived from consensus clustering in the classical workflow for the ovarian cancer case study. (TIF)

S8 Fig Relative changes in area under the CDF curve in the ovarian cancer case study, as the number of clusters (*k*) increase from 2 to 6. When *k* increases from 4 to 5 and so on, the area under the CDF curve does not increase substantially (<0.1), as indicated by the red line.
(TIF)

S9 Fig Gap statistic determined the optimal clustering number. Gap statistic determined the optimal clustering number is k = 4 using the ELM hidden feature(a) and preprocessed gene expression(b) for ovarian cancer.
(TIF)

S10 Fig The four molecular subtypes defined in previous studies showed no significant association with overall survival in ovarian cancer. (TIF)

S11 Fig Survival analysis based on classifications of the validation dataset showed significant association with OS in ovarian cancer. (TIF)

S1 Table Information of datasets. (XLSX)

S2 Table ELM feature matrix for gastric cancer. (XLSX)

S3 Table ELM feature matrix for ovarian cancer. (XLSX)

S4 Table Gene set enrichment analysis for each subtype identified for gastric and ovarian cancers. (XLSX)
