# Correction: Dissecting cancer heterogeneity based on dimension reduction of transcriptomic profiles using extreme learning machines

**DOI:** 10.1371/journal.pone.0205548

**Published:** 2018-10-05

**Authors:** Kejun Wang, Xin Duan, Feng Gao, Wei Wang, Liangliang Liu, Xin Wang

An affiliation for the 6^th^ author is not included. Xin Wang is affiliated with both #2 and #3, Shenzhen Research Institute, City University of Hong Kong, Shenzhen, China.

The following information is missing from the Funding section: The authors acknowledge the support received from Shenzhen Research Institute, City University of Hong Kong.
