# Supplementary figures and images for: Dissecting cancer heterogeneity based on dimension reduction of transcriptomic profiles using extreme learning machines
Source: PLoS One. 2018 Sep 14;13(9):e0203824. doi: 10.1371/journal.pone.0203824 (PMC6138406; doi:10.1371/journal.pone.0203824)

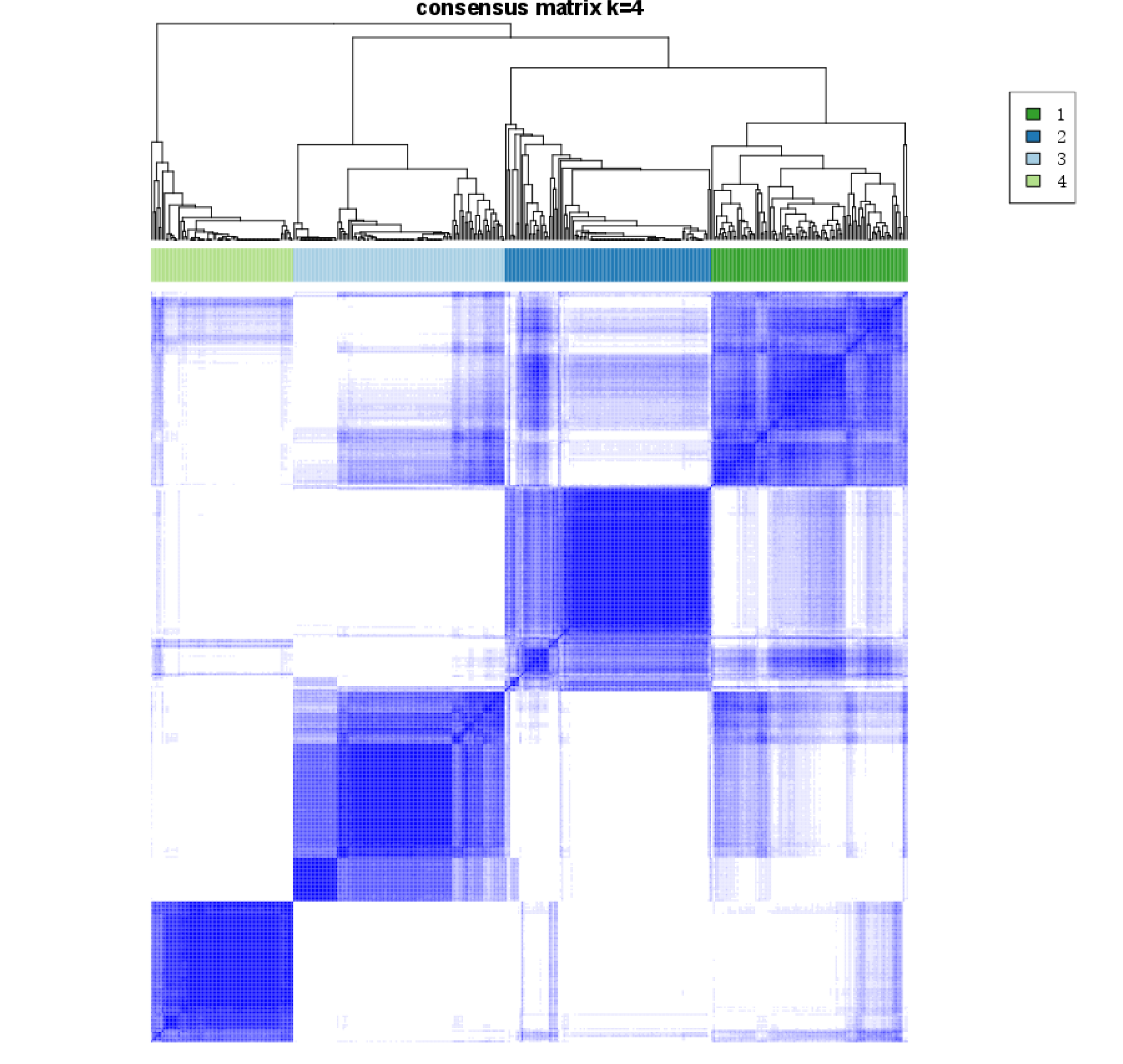

Supplement: S1 Fig — (TIF) [file pone.0203824.s001.tif]

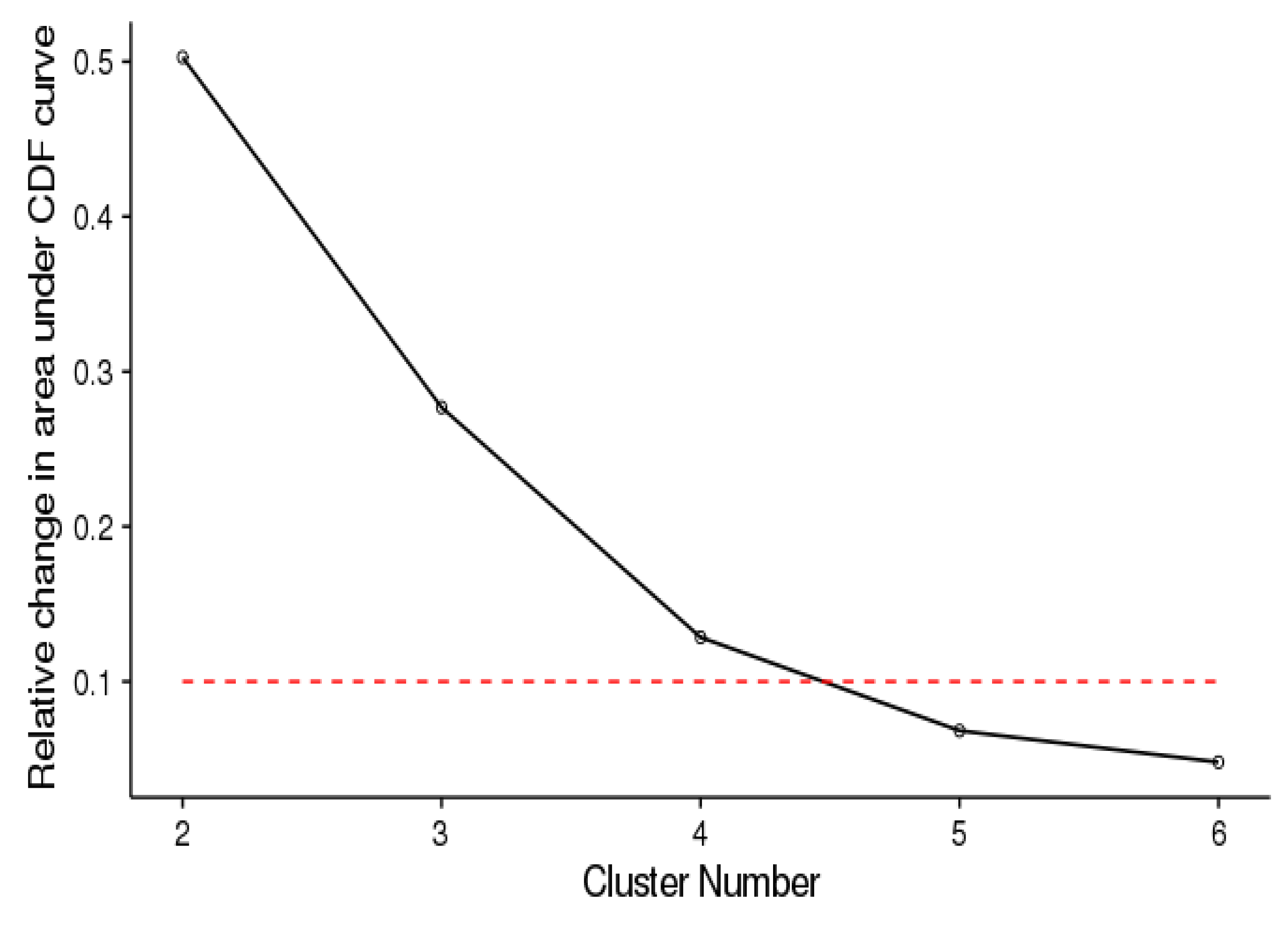

Supplement: S2 Fig — When k increases from 4 to 5 and so on, the area under the CDF curve does not increase substantially (<0.1), as indicated by the red line. (TIF) [file pone.0203824.s002.tif]

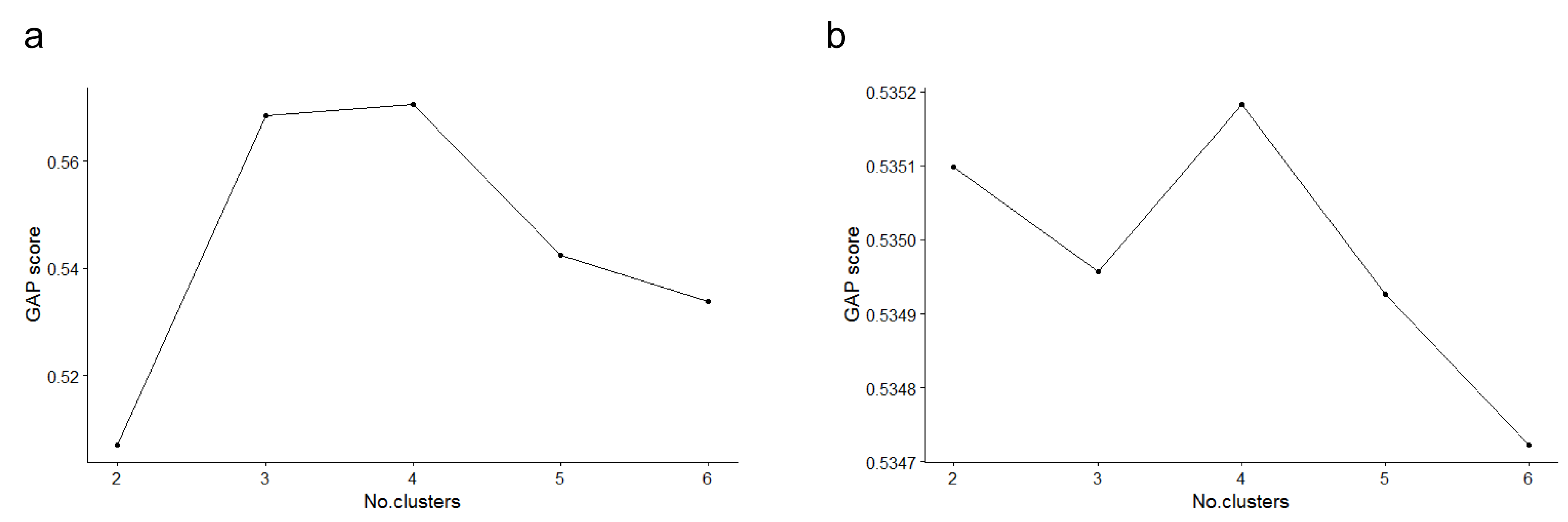

Supplement: S3 Fig — The optimal clustering number is 4 using the ELM hidden feature(a) and preprocessed gene expression(b) for gastric cancer. (TIF) [file pone.0203824.s003.tif]

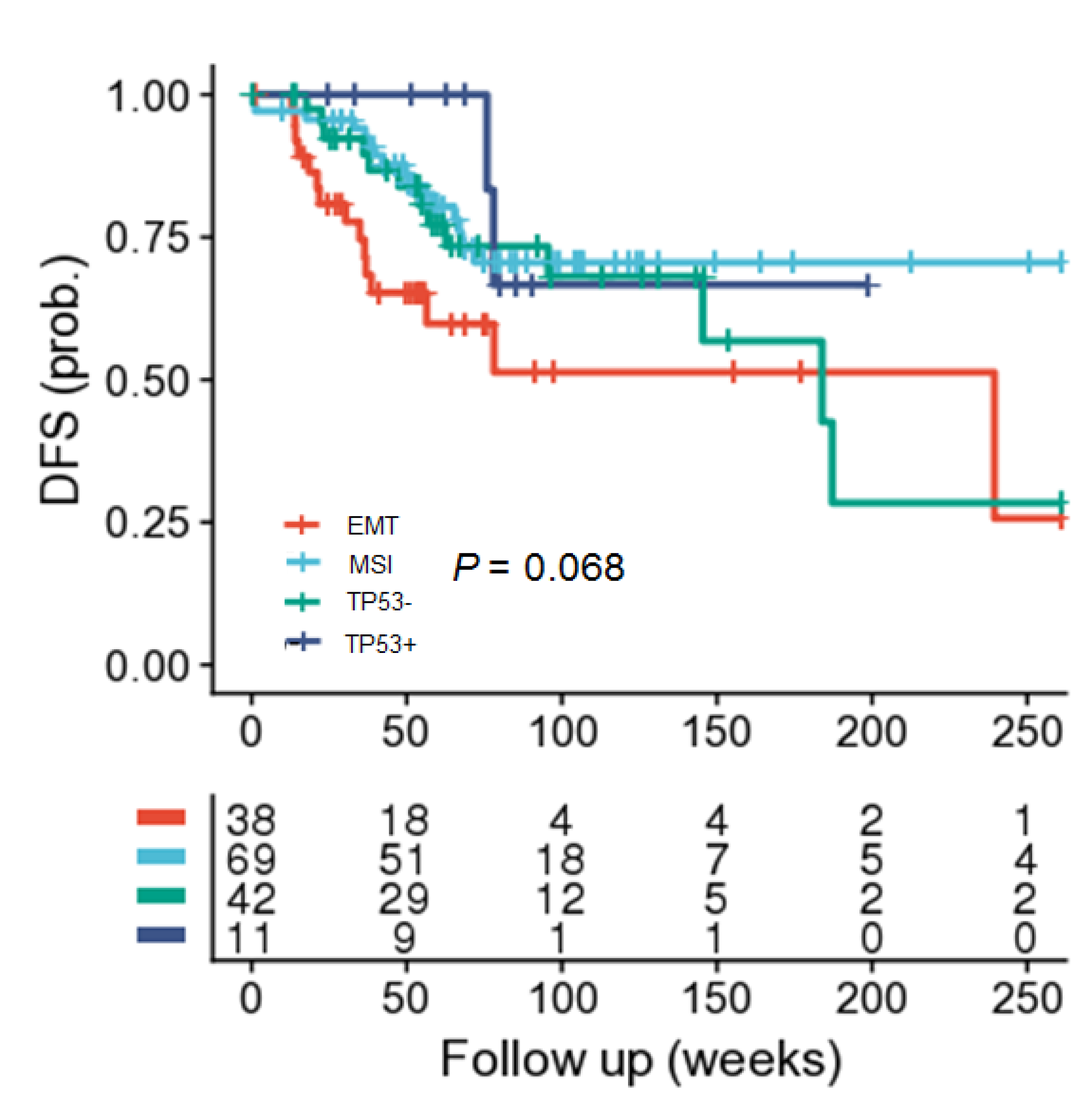

Supplement: S4 Fig — (TIF) [file pone.0203824.s004.tif]

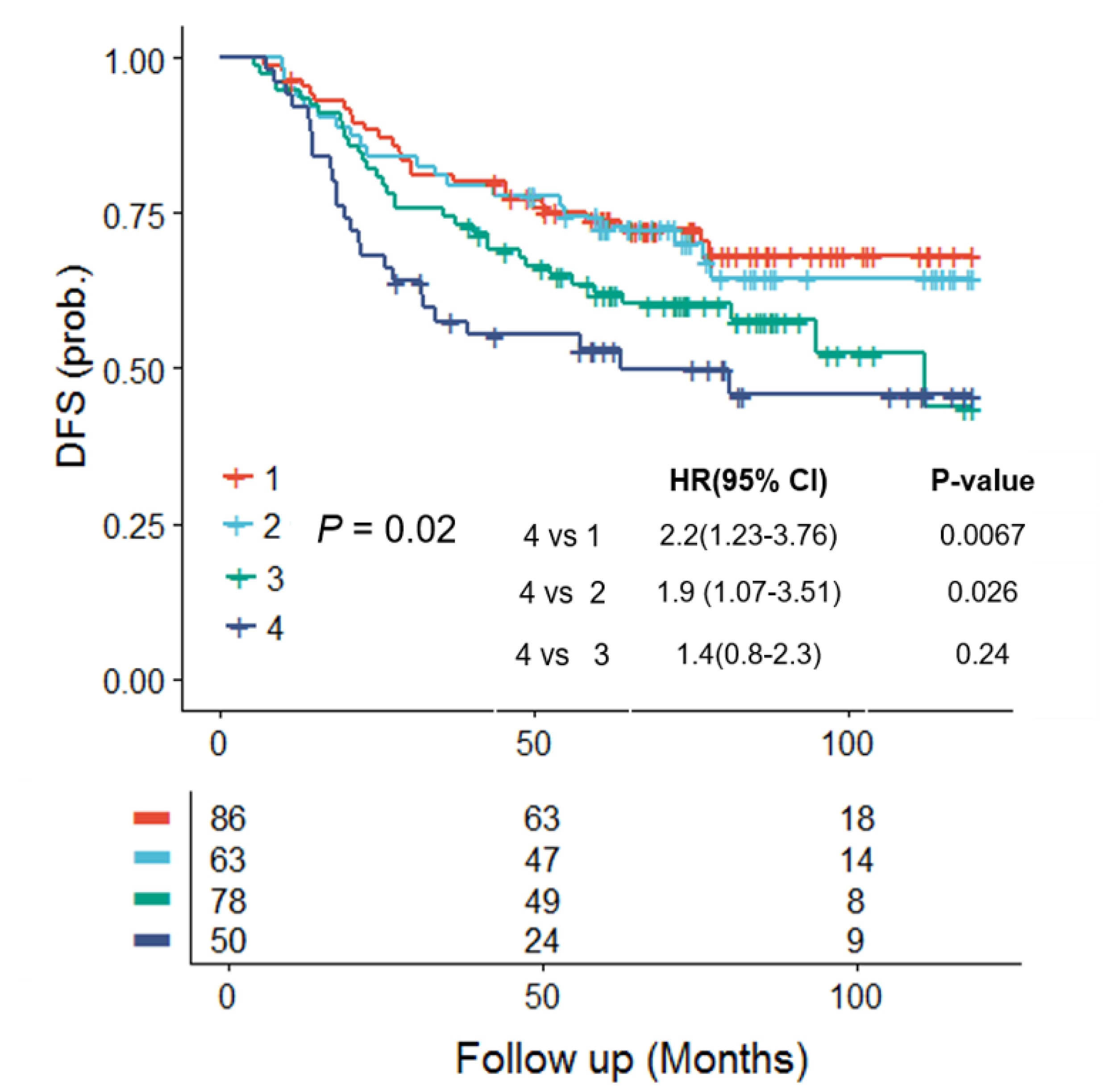

Supplement: S5 Fig — (TIF) [file pone.0203824.s005.tif]

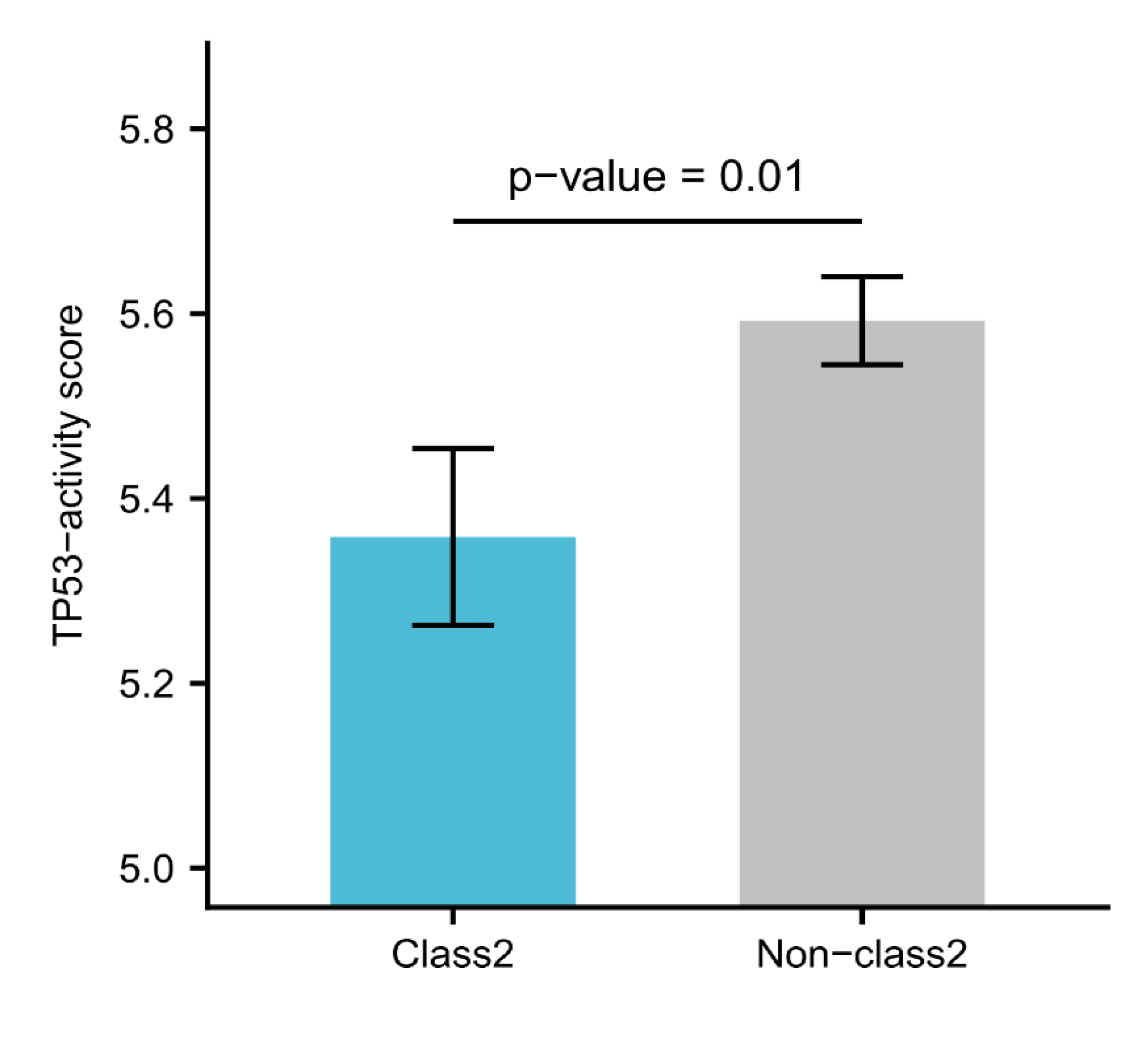

Supplement: S6 Fig — The TP53 activity score was calculated based on average expression levels of CDKN1A and MDM2 genes. (TIF) [file pone.0203824.s006.tif]

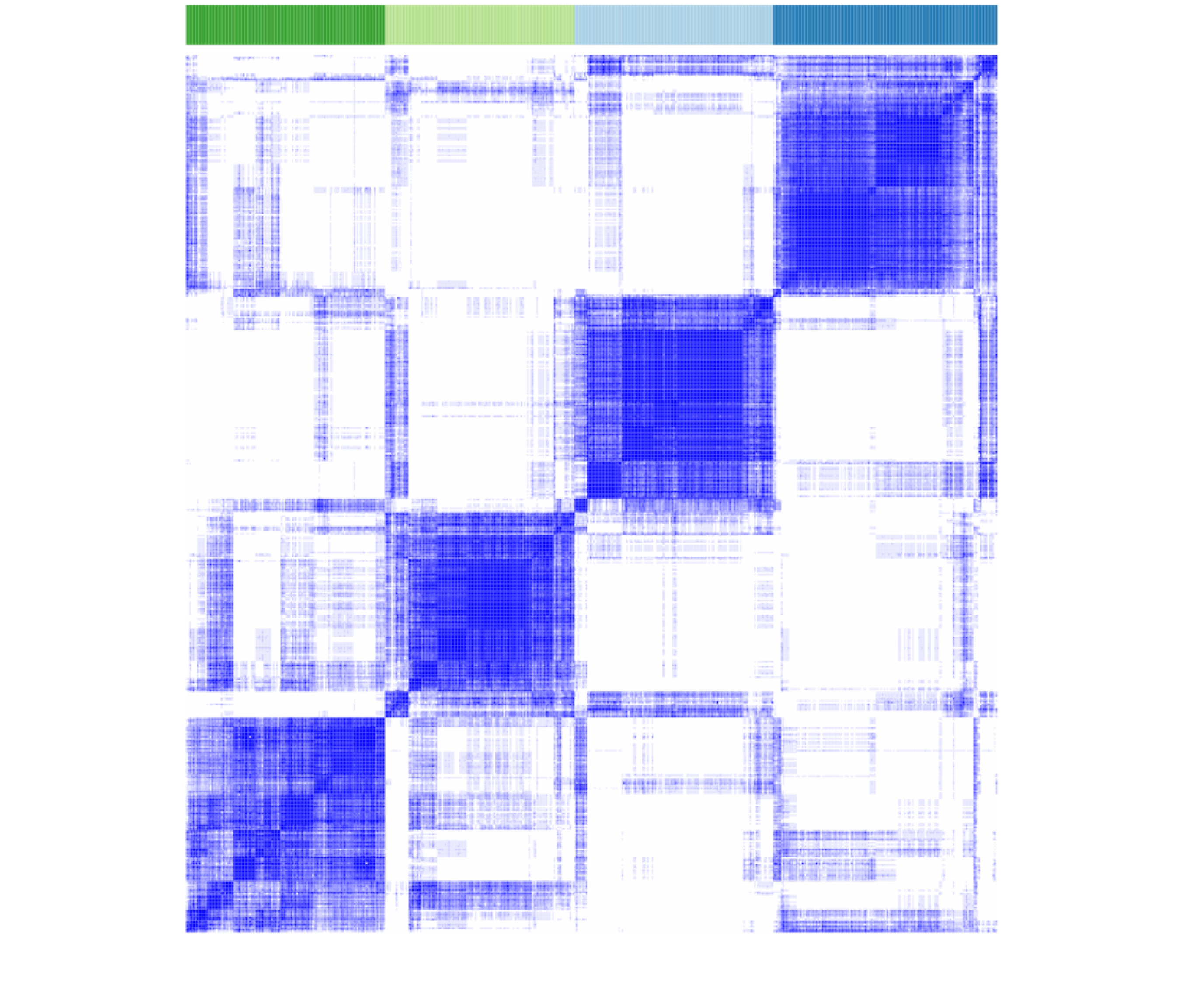

Supplement: S7 Fig — (TIF) [file pone.0203824.s007.tif]

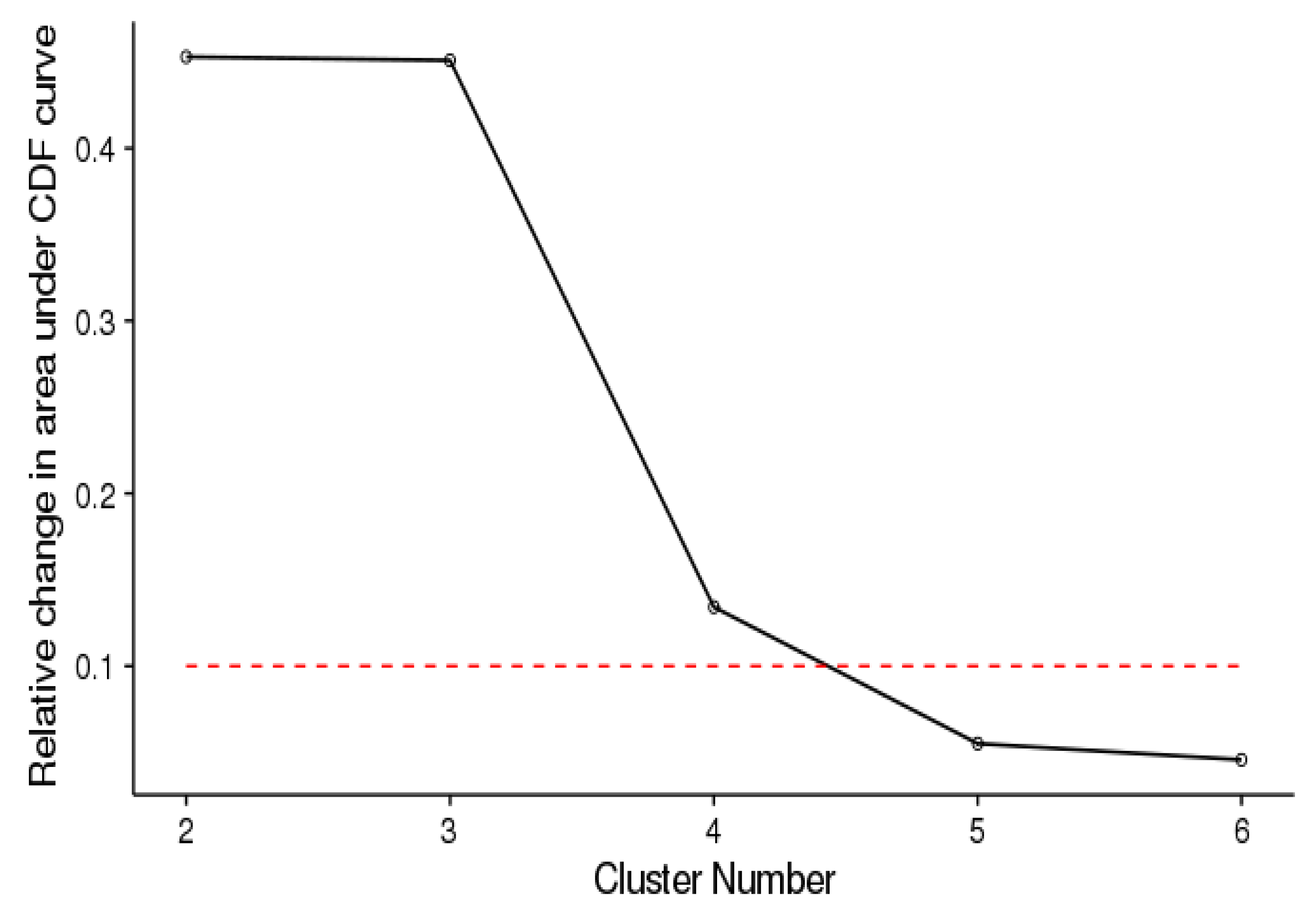

Supplement: S8 Fig — When k increases from 4 to 5 and so on, the area under the CDF curve does not increase substantially (<0.1), as indicated by the red line. (TIF) [file pone.0203824.s008.tif]

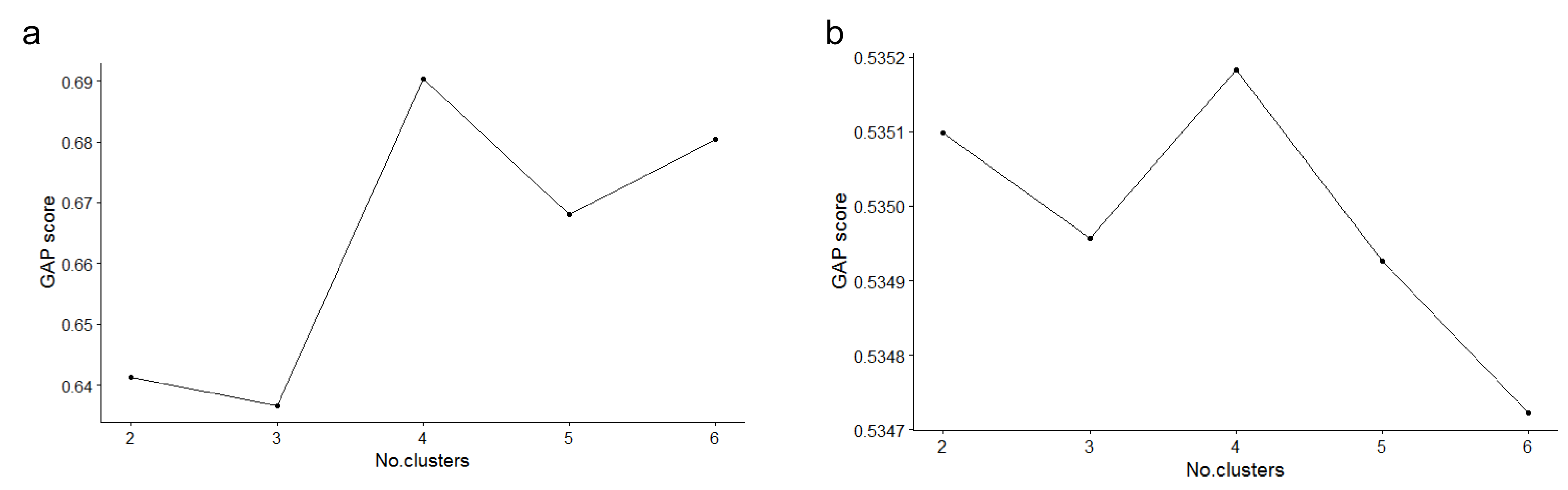

Supplement: S9 Fig — Gap statistic determined the optimal clustering number is k = 4 using the ELM hidden feature(a) and preprocessed gene expression(b) for ovarian cancer. (TIF) [file pone.0203824.s009.tif]

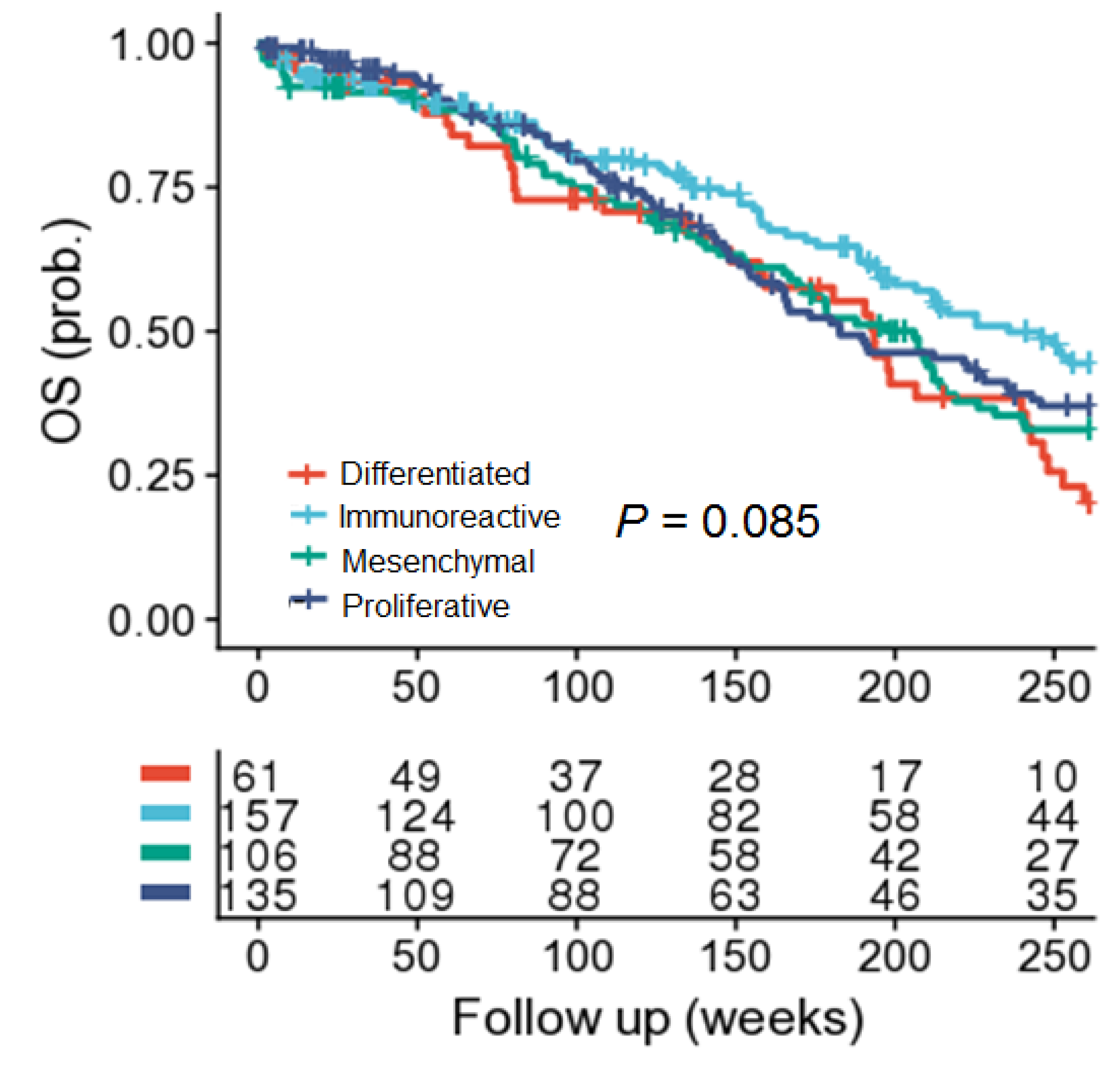

Supplement: S10 Fig — (TIF) [file pone.0203824.s010.tif]

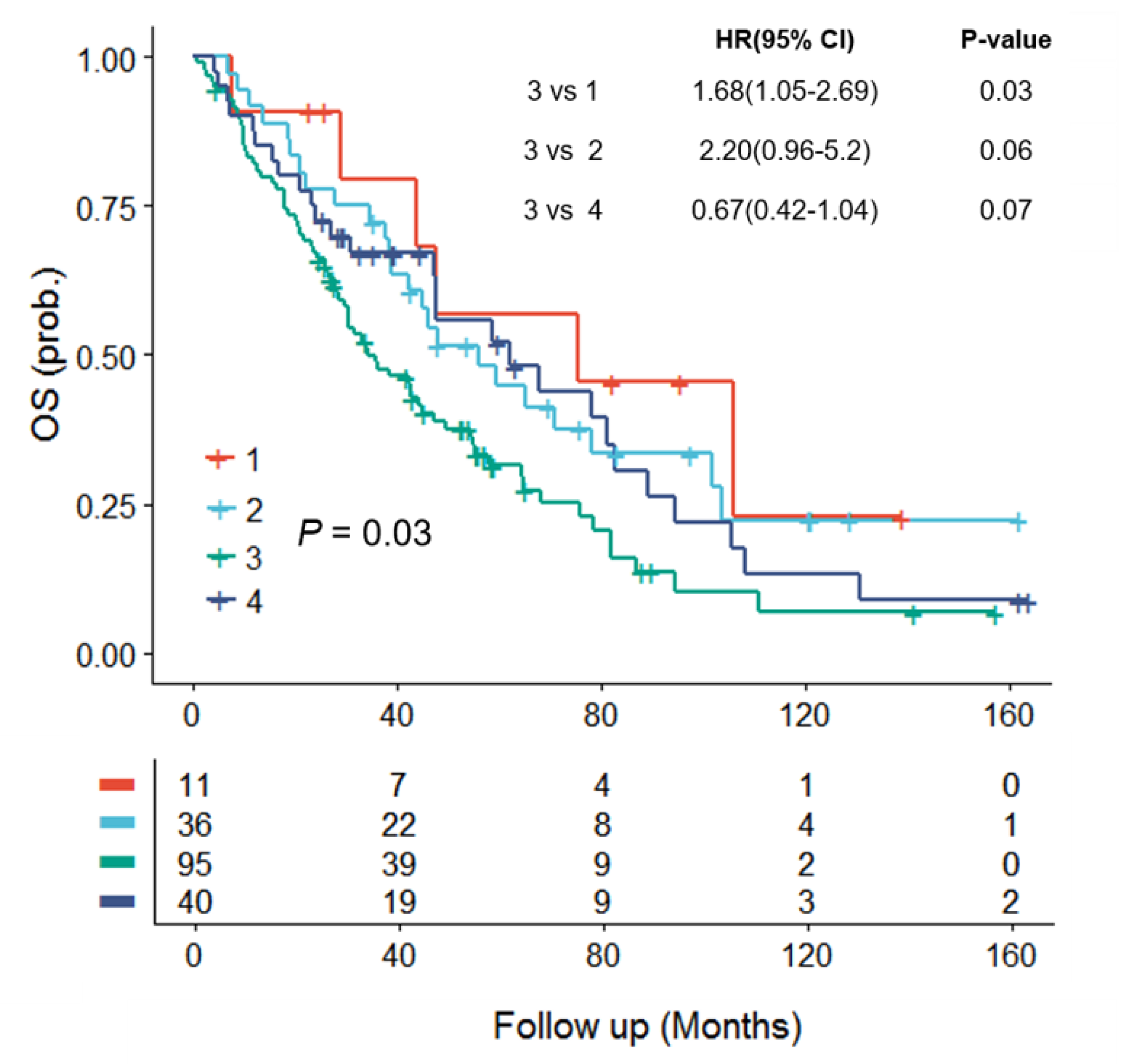

Supplement: S11 Fig — (TIF) [file pone.0203824.s011.tif]
